# PIP5K1A-PIP2-RhoA Signaling Orchestrates Membrane Remodeling during Early Porcine Embryo Development

**DOI:** 10.7150/ijbs.129307

**Published:** 2026-05-15

**Authors:** Cheng-Lin Zhan, Song-Hee Lee, Zheng-Wen Nie, Xiang-Shun Cui

**Affiliations:** 1 Department of Animal Science, Chungbuk National University, Cheongju 28644, Republic of Korea.; 2 Current address: Guangzhou National Laboratory, Guangzhou International Bio Island, Guangzhou 510005, Guangdong, China.

**Keywords:** PIP5K1A, phosphoinositide signaling, calcium oscillation, membrane remodeling, cytoskeletal dynamics

## Abstract

Dynamic plasma membrane remodeling is fundamental for cleavage, signal transduction, and cytoskeletal organization during mammalian embryogenesis. Although oocyte activation triggers membrane remodeling and elevates phosphatidylinositol (4,5)-bisphosphate (PIP2), the underlying regulatory mechanisms remain elusive. Here, we identify phosphatidylinositol-4-phosphate 5-kinase type I alpha (PIP5K1A), an enzyme responsible for generating PIP2, as a key regulator of membrane remodeling in porcine embryos. Maternal depletion of PIP5K1A impaired oocyte activation, disrupted actin organization and vesicle trafficking, and blocked early development. Conversely, excessive expression of PIP5K1A caused accumulation of PIP2-enriched vesicles that trapped filamentous actin and reduced membrane contractility, resulting in cleavage failure. Structure-function analysis revealed that residue Q169 mediates PIP5K1A interaction with RhoA, the PIPB motif anchors it to the plasma membrane, and the activation loop drives catalytic activity and is required for proper RhoA membrane localization and is associated with endocytic abnormalities when mutated. PIP5K1A also sustains phospholipase C-inositol trisphosphate-Ca^2+^ signaling, coordinating vesicle fusion and cytoskeletal remodeling. During cytokinesis, PIP5K1A-enriched membrane clusters serve as hubs for RhoA recruitment. These findings suggest PIP5K1A as a central organizer of lipid signaling, actin dynamics, and membrane contractility to drive successful early embryonic development in mammals.

## Introduction

In early mammalian development, the plasma membrane has a vital role beyond just providing structure, acting as a dynamic interface for signal transduction, cytoskeletal anchoring, and membrane remodeling. Oocytes and cleavage-stage embryos are significantly larger than somatic cells, with proportionally more plasma membrane surface area. During this stage, embryonic transcription is mostly silenced, and cell division depends heavily on maternally deposited transcripts and proteins 1. Therefore, regulation of the plasma membrane and its associated molecular machinery is crucial for proper embryonic development.

Upon fertilization or parthenogenetic activation, oocytes undergo plasma membrane depolarization accompanied by repetitive intracellular Ca^2+^ oscillations 2. These Ca^2+^ transients activate signaling pathways that elevate phosphatidylinositol (4,5)-bisphosphate (PIP2) levels at the membrane, likely through phosphatidylinositol 4-phosphate 5-kinases (PIP5Ks) activity 3. During subsequent cleavage, similar Ca^2+^ signals are maintained, in part through inositol 1,4,5-trisphosphate (IP3)-mediated pathways, and contribute to the formation of new PIP2-enriched membranes in daughter blastomeres 4, 5. These dynamic changes suggest that PIP2 plays a pivotal role in coordinating membrane remodeling and cleavage furrow formation during early embryo development 6.

PIP2 is a phospholipid enriched at the inner leaflet of the plasma membrane, where its negatively charged head groups facilitate interactions with a wide array of proteins 7, 8. PIP2 plays diverse roles in cellular processes, including the organization of the actin cytoskeleton, membrane trafficking, and the establishment of cell polarity 9-11. It directly interacts with key regulators such as cell division cycle 42 (CDC42) 12, RhoA, and anillin to orchestrate polarity signaling and cytokinesis in human cells 13. In addition, PIP2 serves as a precursor for secondary messengers such as IP3 and DAG through hydrolysis by phospholipase C (PLC) 14, 15. PIP2 levels are dynamically controlled by multiple enzymes, including PIP5K1, phosphatidylinositol 5-phosphate 4-kinase type 2 (PIP4K2), and the phosphatase PTEN 16. Among these, PIP5K1A has been identified as a principal contributor to PIP2 synthesis at the plasma membrane in human cells 17.

PIP5K1 enzymes synthesize PIP2 from phosphatidylinositol 4 phosphate (PI4P) and are essential for maintaining membrane phosphoinositide composition 16. Their activity is regulated by small Rho GTPases such as Rac1 and RhoA, which coordinate cytoskeletal dynamics and membrane trafficking 18, 19. Excessive PIP5K1 activity can lead to aberrant membrane structures, including actin-enriched vacuoles 20, and may initiate feed-forward regulatory circuits such as RhoA-PIP5K1-PIP2-RhoA. Similar signaling loops involving phospholipase D (PLD), Ca^2+^ signaling, and PLC-mediated hydrolysis of PIP2 have also been proposed 21. Among the PIP5K1 isoforms, PIP5K1A is particularly well-characterized. It harbors several conserved domains, including a G-rich loop, dimerization motif, 'insert' region, activation loop, and a PIPB motif critical for phosphoinositide binding. Biochemical and simulation studies have shown that the PIPB motif preferentially binds PI4P, while the activation loop displays higher affinity for PIP2 22. These structural features enable PIP5K1A to sense and respond to lipid environments, thereby participating in the regulation of dynamic membranes.

Functional studies on PIP5K1A have identified key residues involved in phosphoinositide binding. For instance, mutation of the PIPB motif (e.g., K238A) or the adjacent GSTYKR segment (e.g., R244A) diminishes the kinase's ability to bind PI4P 22, 23. Similarly, the substitution of lysine residues in the activation loop of PIP5K1B impairs its membrane localization, highlighting the structural sensitivity of these lipid-interacting domains 24. While *in vitro* data have delineated these binding properties, the spatial and temporal dynamics of PIP5K1A-lipid interactions in living embryos remain poorly understood. Moreover, although PIP5K1 has been shown to regulate actin organization in Drosophila oocytes 25, its role in mammalian early embryo development has yet to be fully defined.

Interestingly, PIP2 not only serves as a substrate and product in the PIP5K1-catalyzed reaction but may also modulate the enzyme's activity through direct binding to the activation loop 24. This competition with PI4P suggests a potential positive feedback loop, whereby PIP2 enhances its synthesis through the recruitment and activation of PIP5K1A. While such a mechanism has been proposed *in vitro*, its existence and functional relevance in living embryos remain untested. It was hypothesized that PIP5K1A supports a Ca^2+^- and cytoskeleton-dependent PIP2-PIP5K1A positive feedback loop that drives membrane remodeling and cleavage progression in early porcine embryos. To evaluate this, a combination of gene knockdown, overexpression, domain-specific mutagenesis, and live-cell imaging was employed to dissect the molecular roles of PIP5K1A in membrane dynamics and embryonic cytokinesis.

## Material and Methods

### Experimental design

This study used an *in vitro* parthenogenetic porcine embryo model to examine PIP5K1A function in membrane remodeling and early cleavage. The workflow consisted of oocyte collection, *in vitro* maturation (IVM, 42-44 h), parthenogenetic activation (PA), timed microinjection of dsRNAs (knockdown) or capped mRNAs (overexpression/mutants), and *in vitro* culture (IVC). All developmental stages were timed from activation (hours post-activation, hpa) and confirmed morphologically.

MII oocytes were activated at 0 h, cultured in PZM-5, and microinjected either at the MII stage (pre-activation) or at the 1-cell stage (~6-8 hpa). Embryos were then assessed at fixed time points: 1-cell (~24 hpa), 2-cell (~48 hpa, Day 2), 4-cell (~72 hpa, Day 3), and blastocyst (Day 7). At each time point, embryos were classified as normal (on schedule), delayed (≥1 stage behind but progressing), or arrested (no progression or fragmented).

Within each experiment, oocytes/embryos from the same batch were randomly allocated to groups: control (scrambled dsRNA or vehicle), PIP5K1A knockdown, PIP5K1B knockdown, wild-type PIP5K1A overexpression, and domain-specific mutants (PIPB motif, activation loop, Q169A), with or without fluorescent probes. All experiments were performed in at least three independent biological replicates using different batches of ovaries. Exact embryo numbers per group are indicated in figure legends. Image acquisition and quantification were conducted under identical settings, with blinded analysis whenever possible.

This design enabled direct comparison of loss-of-function, gain-of-function, and structure-function effects at precisely controlled developmental stages while ensuring statistical robustness and reproducibility.

### Oocyte collection and *in vitro* maturation (IVM)

Porcine ovaries were obtained from a local abattoir (Farm Story Dodarm B&F, Umsung, Chungbuk, Republic of Korea) and transported to the lab within 2 h of animal sacrifice in a thermos filled with physiological saline containing 50 mg/mL streptomycin sulfate and 75 mg/mL penicillin G at 30-37 °C. Follicular fluid from follicles 3-6 mm in diameter was aspirated using a 12-gauge needle attached to a 10 mL disposable syringe. Cumulus-oocyte complexes (COCs) with uniform cytoplasm and three or more layers of cumulus granulosa cells were collected under a stereomicroscope for use in further experiments. The IVM medium was ICM-199 (Invitrogen, 11150-059) supplemented with 100 mg/L sodium pyruvate, 10 ng/mL epidermal growth factor, 10% (v/v) porcine follicular fluid, 10 IU/mL follicle-stimulating hormone, and 10 IU/mL luteinizing hormone. After washing thrice with balanced IVM, approximately 80 COCs were transferred to each well of a 4-well plate (SPL Life Sciences, 30004). The plate was then covered with mineral oil (370 μL/well) and incubated at 38.5 °C for 42-44 h in an atmosphere of 5% CO_2_ and 100% humidity.

### Parthenogenetic activation (PA) and *in vitro* culture (IVC)

For membrane-remodeling analyses, parthenogenetic activation (PA) was used to provide synchronized Ca^2+^-triggered entry into development and minimize sperm-derived variability 26. After 44 h of IVM, COCs with extended cumulus cells were pipetted 20-30 times in a solution of 1 mg/mL hyaluronidase to remove cumulus cells. Oocytes at the MII stage, exhibiting the first polar body, were selected and parthenogenetically activated by two direct-current pulses of 120 V for 60 µs in 297 mM mannitol (pH 7.2) containing 0.01% polyvinyl alcohol (PVA, w/v), 0.5 mM HEPES, 0.05 mM MgSO_4_, and 0.1 mM CaCl_2_. The activated oocytes were then cultured in bicarbonate-buffered porcine zygote medium 5 (PZM-5) containing 7.5 µg/mL cytochalasin B and 5 mg/mL BSA for 3 h to suppress the extrusion of the pseudo-second polar body. After thorough washing, the oocytes were then transferred to *in vitro* culture medium (bicarbonate-buffered PZM-5 supplemented with 5 mg/mL BSA) and cultured in a 5% CO_2_ incubator at 38.5 °C.

### RNA extraction, reverse transcription, and quantitative polymerase chain reaction (qPCR)

Messenger RNA (mRNA) was extracted from 50 embryos per group using a Dynabeads mRNA DIRECT Purification Kit (Thermo Fisher Scientific, 61012) according to the manufacturer's instructions. cDNAs were obtained by reverse transcription of mRNA using the Express 1st Strand cDNA Synthesis System Kit (LeGene, 6210-20) and amplified using the WizPure qPCR Master with Super Green Kit (Wizbio Solutions, W1731-8). The amplification cycles were as follows: 95 °C for 5 min, followed by 40 cycles of 95 °C for 15 s, annealing for 25 s, 72 °C for 10 s, and final extension at 72 °C for 10 min. Gene expression levels were normalized to that of GAPDH (internal control) for knockdown experiments and GFP (outer control) for comparisons between different developmental stages. Relative quantification was performed using the 2^-ΔΔCT^ method. The sequences of all primers and their annealing temperatures used for RT-PCR are provided in Table S1.

### Immunofluorescent staining and confocal microscopy

Embryos were promptly fixed in 3.7% formaldehyde for 1 h, followed by three washes with PBS/polyvinyl alcohol (PBS/PVA), permeabilized with PBS/PVA containing 1% Triton X-100 for 1 h, washed again with PBS/PVA, and blocked with 1.0% BSA in PVA/PBS for 1 h. For immunostaining, embryos were incubated overnight at 4 °C with an anti-anillin antibody or stained with phalloidin for 1 h. Embryos were incubated at 37 °C with the secondary antibody for 1 h after another PBS/PVA wash and subsequently stained with bisBenzimide H33342 trihydrochloride (Sigma-Aldrich, B2261, 1:2000) for 15 min, followed by another PBS/PVA wash. The prepared embryos were mounted onto slides and examined using a confocal microscope (Zeiss LSM 710 META, Jena, Germany). Image processing was conducted using Zen software (version 8.0; Zeiss, Jena, Germany).

### Vector construction and the production of *in vitro* RNAs

Plasmids, including mCherry-UtrCH and tubulin-eGFP, were purchased from Addgene (Addgene, USA). RNAs were extracted from the cumulus cells of 50 metaphase II (MII) oocytes and 1-cell embryos, respectively, from which cDNAs were synthesized using RT-PCR. DNA fragments, including the PH fragments of *PLCD1*,* PLEKHA3,* and *GRP1*, and the gene-coding sequence of* PIP5K1A,* were amplified using Phusion high-fidelity polymerase (Thermo Scientific, USA). DNA fragments were inserted into a modified pCS2+ vector (Addgene, USA). For eGFP, mCherry, PH_PLCD1_-eGFP, PH_PLEKHA3_-mCherry, PH_GRP1_-mCherry, and PIP5K1A-mCherry, plasmids containing only a mCherry or eGFP label, and vectors fused to the DNA fragments with a mCherry or eGFP label at the N-terminus were constructed using restriction enzymes and a DNA ligase. For mutated *PIP5K1A*, vectors with point-mutated DNA fragments-mCherry or -eGFP labels were constructed using the AccuRapid Cloning Kit (Bioneer, USA). All the vectors were linearized as transcriptional templates using restriction enzymes. Templates for *PIP5K1A*, *PIP5K1B,* and* PTEN* dsRNAs were produced using PCR based on gene coding sequences with an Ampmaster Taq kit (Geneall, South Korea). All *in vitro* mRNAs and dsRNAs were transcribed using mMessage mMachine^TM^ SP6 (Thermo Fisher Scientific, USA) or T3 (Thermo Fisher Scientific, USA) kits, purified using the Riboclear^TM^ Kit (Geneall, South Korea), and eluted in RNase-free water. The sequences of all primers used to produce mRNAs and dsRNAs are provided in Table S1.

### Microinjection

MII oocytes and one-cell stage parthenogenetic embryos were microinjected with RNAs under a Nikon TE2000-U inverted microscope (Nikon Corporation, Tokyo, Japan) using a FemtoJet microinjector (Eppendorf, Hamburg, Germany). The microinjected RNAs at different concentrations and their purposes are shown in Table S2. To ensure actin dynamics reflected endogenous biology, we established a minimal-perturbation threshold using the lowest mRNA concentration that yielded a reliable signal-to-noise ratio. Embryos injected at this dose showed cleavage timing and development rates indistinguishable from non-injected controls. High-dose conditions inducing actin bundling or cytokinesis failure were excluded from mechanistic analysis and used only for contrastive purposes.

### Live cell imaging

Live oocytes or embryos were digested using hyaluronidase until the zona pellucida was soft and deformed, incubated for 10 min, and centrifuged at 12,000 rpm for 20 min, washed three times, and incubated for 2.5 h in PZM-5 containing 7.5 μg/mL cytochalasin B at 38.5 °C. The zona pellucida and extracellular lipid droplets were thoroughly removed using hyaluronidase, followed by repeated pipetting. Denuded oocytes or embryos were put into a drop containing 5 μL PZM-5 and covered with mineral oil at the center of a confocal-imaging dish. Mineral oil was covered with a cover glass and gradually removed until the oocytes or embryos were squeezed. The fluorescence of the oocytes or embryos was measured using an NIS-element live-cell imaging system (Nikon Corporation, Tokyo, Japan). For live-imaging analyses, we used the following descriptive terms based on size, morphology, cortical localization, and marker co-labeling (PH_PLCD1_ for PIP2, UtrCH for F-actin, and VAMP2 for vesicles): dots (< 1 μm cortical puncta), bubbles (> 2 μm spherical vesicular structures emerging from the cortex/plasma membrane), and plaques (≥2 μm relatively flat cortical domains). We refer to large rounded PH_PLCD1_/UtrCH co-positive cytoplasmic aggregates as PIP2-actin nuclei and to star-like radial F-actin arrays emanating from a PH_PLCD1_-positive cortical focus as PIP2-actin astral.

### PIP2 content measurement

The zona pellucida was removed from oocytes or embryos by brief incubation in 0.5% pronase solution. Lipids were extracted from 200 embryos per group. Briefly, embryos were washed three times in ice-cold PBS/PVA and lysed in 0.5 M trichloroacetic acid (TCA) on ice. After centrifugation, the pellet was washed twice with 5% TCA/1 mM EDTA. Neutral lipids were removed by two extractions with methanol: chloroform (2:1, v/v). Acidic lipids were then extracted with methanol: chloroform:12 M HCl (80:40:1, v/v) for 25 min at room temperature. Following phase separation by addition of chloroform and 0.1 M HCl, the organic phase was collected and dried under vacuum. The dried lipids were resuspended in 125 μL PBS + 0.25% Protein Stabilizer (provided in the Echelon kit). Each sample was analyzed in duplicate using the PI(4,5)P2 Mass ELISA Kit (Echelon Biosciences Inc, K-4500) according to the manufacturer's instructions. Absorbance was measured at 450 nm, and PIP2 content was calculated from the standard curve and normalized to embryo number.

### Western blot

For the whole cell samples, 150 embryos of each group were collected in 20 μL SDS sample buffer. For membrane and cytoplasmic samples, the cell fractions of embryos were separated using a modified method 27, and 300 embryos from each group were collected and sonicated for 10 min. The obtained cell fractions were centrifuged at 1200 × g for 10 min, and the pellet was collected as sample P1. The supernatant was centrifuged for 1 h at 120,000 × g, and the pellet and supernatant were collected as P2 and S samples, respectively. The same volume of the sample was resuspended in SDS sample buffer. All samples were denatured at 98 °C for 10 min. Proteins were separated using sodium dodecyl sulfate-polyacrylamide gel electrophoresis and transferred to polyvinylidene fluoride membranes. The membranes were blocked in 5% skim milk in TBS for 1 h, incubated at 4 °C overnight with mouse anti-PIP5K1A (1:1000, Santa Cruz, sc-376634), mouse anti-β-actin (1:1000, Santa Cruz, sc-5274), rabbit anti-anillin (1:1000 Sigma, HPA050556), rabbit anti-Phospho-PLCγ1 (Tyr783) (1:1000, Cell Signaling, 2821T) or mouse anti-RhoA (1:1000, Santa Cruz, sc-418) antibodies, washed three times with TBS containing 0.1% Tween 20, incubated with peroxidase-conjugated goat anti-rabbit or anti-mouse IgG (1:20000, GeneTex, GTX213110-01, GTX213111-01) for 1 h, and detected using Pierce ECL Western blotting substrate (Thermo Fisher Scientific, 32106).

### Active Rho pull-down and detection

The active Rho protein was detected using the Active Rho Pull-Down and Detection Kit (Thermo Fisher, 16116) following the manufacturer's instructions. Briefly, 200 embryos in each group were lysed after removing the zona pellucida. The lysates were incubated with Glutathione Resin coated with GST-Rhotekin-RBD at 4 °C for one hour with gentle rocking. The eluted products were analyzed by Western blot to assess their expression levels.

### Co-Immunoprecipitation

Co-immunoprecipitation (Co-IP) was performed using the Pierce Co-Immunoprecipitation Kit according to the manufacturer's instructions (Thermo Scientific, 26149). Briefly, 400 embryos in each group were lysed after removing the zona pellucida. The lysates were incubated with the antibody-coupled resin at 4 °C overnight with gentle rocking. The eluted products were analyzed by Western blot to assess their expression levels.

### IP3 content detection

The levels of IP3 were determined using the IP3 Competitive ELISA Kit (Invitrogen, EEL210) according to the manufacturer's instructions. For each group, lysates from 300 two-cell embryos were used for the assay.

### Intercellular Ca^2+^ fluorescence staining

After 24 hours of IVC, 2-cell stage embryos were collected for Ca^2+^ fluorescence staining. Embryos were incubated with the Fluo-4, AM, cell-permeant dye (Invitrogen, F14217) in a 37 °C incubator for 1 hour. After washing three times with PBS/PVA, the fluorescence was visualized using a fluorescence microscope. Fluorescence intensity was analyzed using ImageJ.

### Statistical analysis

ImageJ software was used to analyze the membranous and cytosolic fluorescent intensities. For fluorescence analysis at the membrane and other special locations, interesting regions were targeted by drawing lines, and the plot profile data were obtained. The cytosolic distribution of fluorescent fusion proteins was analyzed according to previously published methods 28. All data except fluorescent distribution in graphs are shown as mean ± SEM. Statistical analyses were performed using the Statistical Package for the Social Sciences software (IBM SPSS Statistics 25). For comparisons between two treatment groups, a two-tailed Student's t-test was employed. For comparisons involving multiple groups, one-way analysis of variance (ANOVA) was conducted, followed by the least-significant difference (LSD) test to determine specific inter-group differences. The sample size 'n' is defined as the total number of embryos analyzed across at least three independent biological replicates. Differences were considered significant if P < 0.05, and highly significant if P < 0.01.

## Results

### Remnant PIP2-F-actin vesicles persist in cleavage-arrested porcine embryos

**Figure 1:**
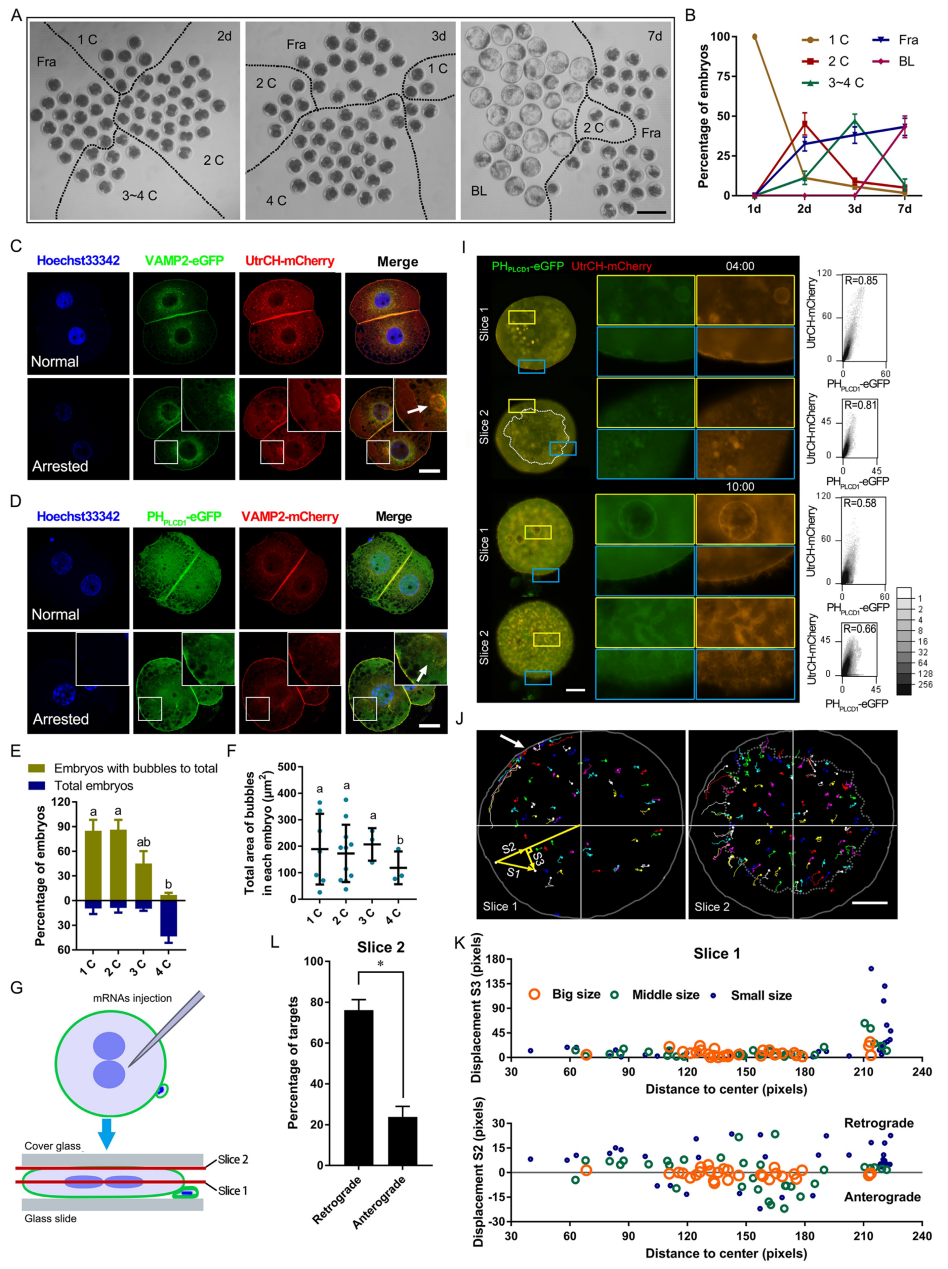
Pattern of PIP2 synthetases expression and localization.** (A)** Early porcine embryo development at days 2, 3, and 7, showing 1-cell (1C), 2-cell (2C), 3-cell (3C), 3-4-cell (3~4C), fragmented (Fra), and blastocyst (BL) stages. Scale bar = 100 μm.** (B)** Percentage of embryos at each developmental stage.** (C)** Co-localization of VAMP2-eGFP and UtrCH-mCherry in normal (stage matches day) and arrested 2-cell embryos, with arrows indicating bubbles. Scale bar = 25 μm.** (D)** Co-localization of PH_PLCD1_-eGFP and VAMP2-mCherry in normal and arrested 2-cell embryos, with arrows marking bubbles. Scale bar = 25 μm.** (E)** Ratio of total embryos to bubble-containing embryos at each stage on day 3.** (F)** Total bubble area in embryos at each stage on day 3.** (G)** Schematic of flattened oocytes/embryos for live imaging, scanning center (slice 1) and top (slice 2) layers.** (H)** Live imaging of PH_PLCD1_-eGFP and UtrCH-mCherry in embryos 4 h and 10 h after CB treatment on day 1. The outer area of the dotted line in the embryo indicates the dynamic plasma membrane and cortex. Scale bar = 25 μm. Right graphs: Pearson's correlation coefficient for PH_PLCD1_-eGFP and UtrCH-mCherry.** (I)** Targeting and tracking of dots and bubbles with PH_PLCD1_-eGFP and UtrCH-mCherry 4 h after CB treatment (Fig. 1H). All tracks are displayed in different colors, where lines represent the moving line and dots indicate its endpoints. Gray solid line, shapes of embryos; the Outer area of the gray dotted line in the embryo showed the plasma membrane and cortex. White arrow, aggregate point. Yellow straight lines S1, S2, and S3 are the actual, vertical (retrograde and anterograde), and horizontal track displacements, respectively.** (J)** S2 and S3 of different-sized targets at different original positions from the center. **(K)** Percentage of retrograde and anterograde targets 4 h after CB treatment. “*” showed a significant difference.

To examine membrane dynamics during early development, porcine embryos were cultured *in vitro* for six days. A subset of embryos exhibited developmental arrest or delay (Figure 1A, B). In arrested 2-cell embryos, prominent vesicle-like structures labeled by vesicle-associated membrane protein 2 (VAMP2)-mCherry were observed. These structures resembled plasma membrane-derived bubbles (Figure 1C). Co-expression of PH_PLCD1_-eGFP (Phospholipase C Delta 1, a specific probe for PIP2) 29 and UtrCH-mCherry (mCherry-fused calponin homology domain of utrophin, a marker for membrane-targeted F-actin) revealed that these vesicle-like bubbles were enriched in both PIP2 and F-actin (Figure 1C, D). For all vesicle quantification analyses, only morphologically confirmed 2-cell embryos were included to avoid stage-dependent bias. An increasing number of bubbles co-localized with PH_PLCD1_-eGFP and UtrCH-mCherry were found in the cytoplasm of arrested or delayed embryos (Figure 1E, F), suggesting an association with defective cytokinesis.

Live-cell imaging further revealed dynamic membrane remodeling. Following oocyte activation, PIP2- and actin-associated signals redistributed from the plasma membrane into the cytoplasm, and vesicle-like structures rapidly formed after removal of cytochalasin B (CB, an inhibitor of F-actin) (Figure S1A, B; Movie S1A).

To enhance visualization, embryos were mechanically flattened and subjected to time-lapse confocal microscopy (Figure 1G). During live imaging, PIP2- and actin-enriched vesicles in the cortex were observed to migrate toward a polar point at the plasma membrane (Figure 1H-K). Overexpression of UtrCH-mCherry induced fibers with PH_PLCD1_-eGFP to cluster at the outer surface of the plasma membrane, which disrupted cytokinesis (Movie S1B, C). In contrast, at physiological expression levels during cytokinesis, the PIP2-actin aggregates moved to the cleavage furrow along the plasma membrane and formed a huge astral. The furrow deepened astrally at the top, and fibers with less UtrCH-mCherry at the outer surface of the plasma membrane gradually grew (Movie S1D). A similar PIP2-actin astral was also formed and trapped in the cleavage furrow of fragmented embryos (Movie S1E); however, their overgrowth did not promote furrow invagination (Movie S1F).

Together, these findings suggested that F-actin and VAMP2 promoted PIP2 nucleation at the plasma membrane. Proper localization and expression of these components were essential for cleavage furrow formation.

### PIP2 synthesis in early embryos is primarily mediated by maternally expressed PIP5K1A

**Figure 2:**
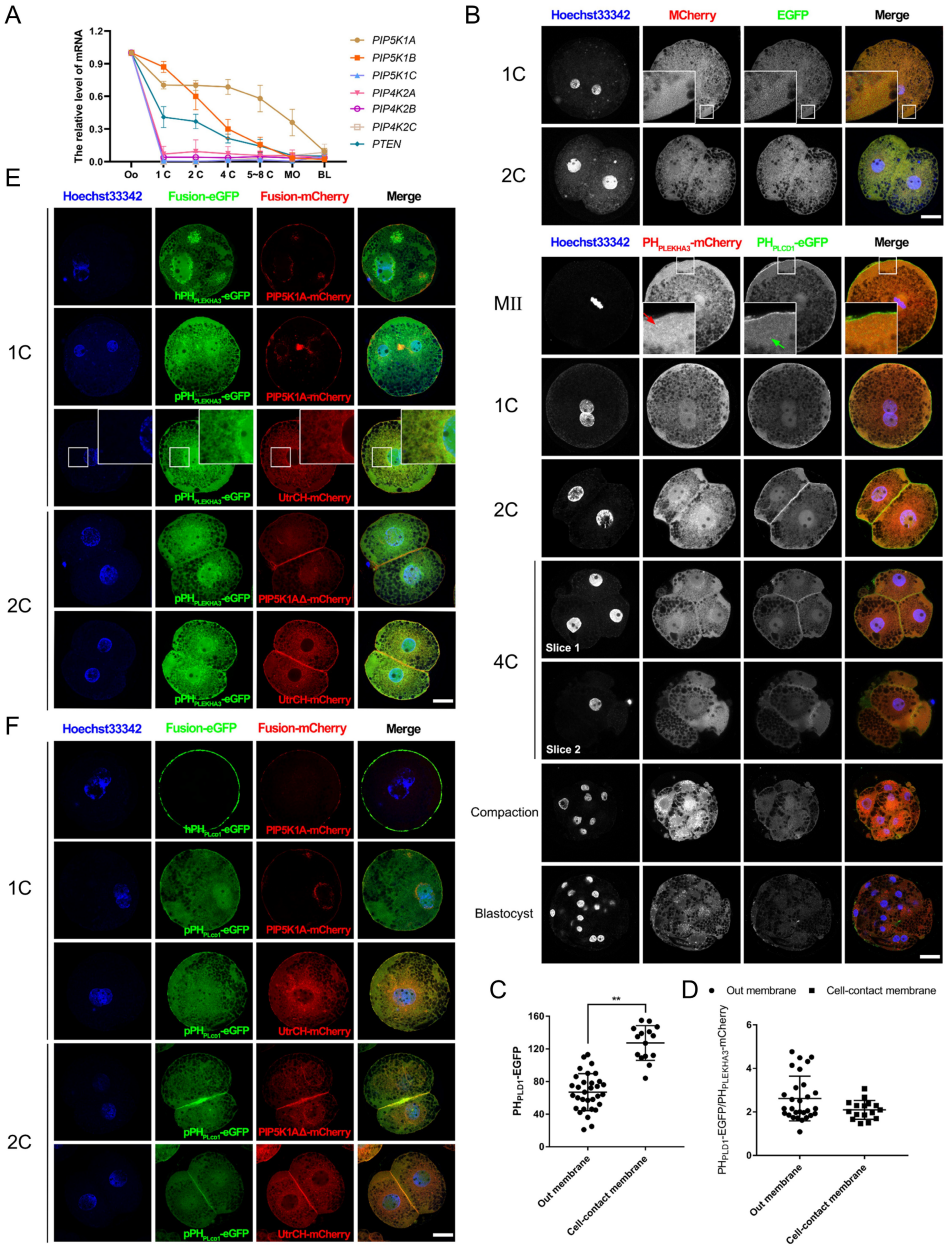
Pattern of PIP2 synthetases expression and localization.** (A)** Relative mRNA levels of PTEN, PIP5K1A, and PIP5K1B during early porcine embryonic development. Data were shown as mean ± SEM. Three replicates were used for each stage. The oocyte count was set to 1.** (B)** Subcellular localization of PH_PLEKHA3_-mCherry (PI4P marker) and PH_PLCD1_-eGFP (PIP2 marker) during early porcine embryonic development. The subcellular localization of mCherry and eGFP was used as a control. Red and yellow arrows represent PI4P and PIP2 dots, respectively.** (C)** The relative intensity of PH_PLCD1_-eGFP in 2-cell embryos.** (D)** The ratio of the intensity of PH_PLCD1_-eGFP to PH_PLEKHA3_-mCherry in 2-cell embryos.** (E)** Co-localization of PH_PLEKHA3_-mCherry, UtrCH-mCherry, and PIP5K1A-mCherry in porcine embryos.** (F)** Co-localization of UtrCH-mCherry, PH_PLCD1_-mCherry, and PIP5K1A-mCherry in porcine embryos. Scale bar = 25 μm. Oo, oocyte; MO, morula; BL, blastocyst. Prefix letters of PH_PLEKHA3_-mCherry and PH_PLCD1_-mCherry: “h,” human; “p,” porcine.

To identify the source of membranous PIP2, we first examined the expression of key phosphoinositide regulators. Among these, *PTEN*, *PIP5K1A*, and *PIP5K1B* were maternally expressed, with *PIP5K1A* showing the most sustained expression up to the morula stage (Figure 2A). Subcellular localization analysis revealed distinct distributions of phosphoinositide species. The PIP3 sensor PH_GRP1_-mCherry (General Receptor for Phosphoinositide 1) and the PIP2 probe PH_PLCD1_-eGFP were largely co-localized in the nucleus and spindle regions (Figure S2A, B). Consistently, immunostaining showed that PTEN, which converts PIP3 to PIP2 30, was enriched at the spindle, whereas PIP5K1A was not (Figure S2C, D), suggesting that PTEN-derived PIP2 is primarily associated with nuclear and mitotic structures.

In contrast, the PI4P sensor PH_PLEKHA3_ (Pleckstrin Homology Domain Containing A3)-mCherry 29 was presented in the cytoplasm and nucleus, with increased intensity near the nuclear envelope as development progressed. In contrast, PH_PLCD1_-eGFP localized predominantly at the plasma membrane and nucleus but lost specific membrane enrichment after compaction (Figure 2B). Notably, PH_PLCD1_-eGFP was significantly enriched at cell-contact sites (Figure 2C). The ratio of the signal intensity of PH_PLCD1_-eGFP to PH_PLEKHA3_-mCherry did not differ between the outside and cell-contact membranes (Figure 2D).

During early cleavages stages, PIP2 was strongly enriched at the cleavage furrow, spindle midzone, and plasma membrane, particularly at intercellular junctions (Figure S3A-C). Colocalization analysis further showed that PIP2 and PIP5K1A were associated with membrane-localized F-actin, whereas PI4P remained primarily perinuclear (Figure 2E, F). These results suggest that PTEN-produced PIP2 engaged in nuclear function or division, whereas PIP5K1A-produced PIP2 participated in plasma membrane function or cytokinesis.

### PIP5K1A knockdown impairs PIP2 production, oocyte activation, and early embryonic development

**Figure 3:**
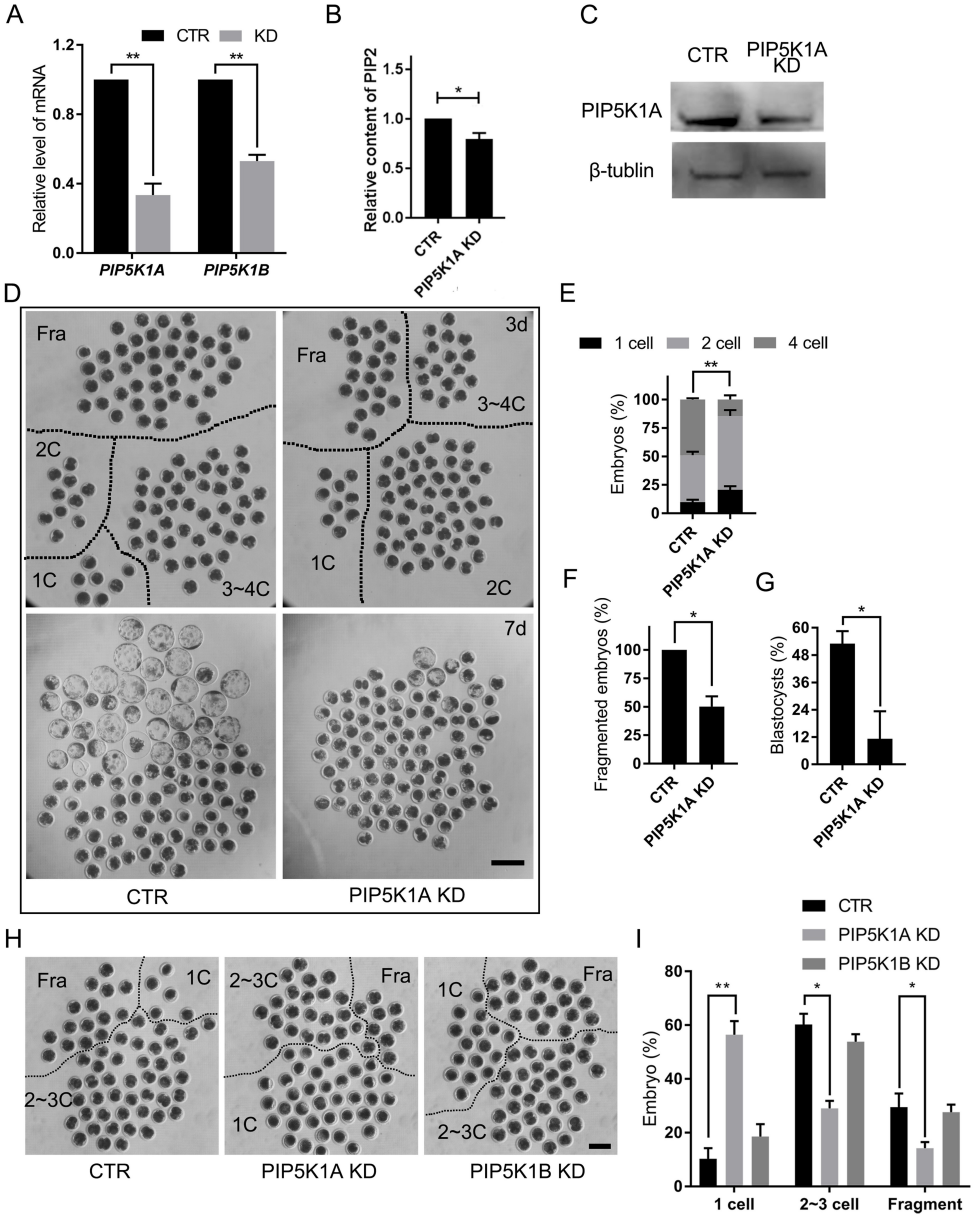
Effects of PIP5K1A knockdown on oocyte activation and embryo development. **(A)** mRNA levels of PIP5K1A and PIP5K1B after knockdown in porcine embryos on day 2. Data were shown as mean ± SEM. Three replicates were used for each stage.** (B)** PIP2 level after PIP5K1A knockdown in porcine embryos at Day 2.** (C)** Protein levels of PIP5K1A after knockdown in porcine embryos on day 2.** (D)** Porcine embryonic development on days 3 and 7. Scale bar = 300 μm.** (E)** Ratio of embryos at different stages on day 3.** (F)** Ratio of fragmented embryos on day 3.** (G)** The ratio of blastocysts on day 7.** (H)** Porcine oocytes at 1 d after parthenogenetic activation. Scale bar = 200 μm.** (I)** The ratio of embryos 1 d after parthenogenetic activation.

To assess the functions of the PIP5K1 isoform, dsRNAs targeting PIP5K1A or PIP5K1B were microinjected into porcine oocytes or 1-cell embryos (Figure 3A). Only PIP5K1A knockdown resulted in significantly decreased PIP2 concentrations in embryos (Figure 3B). Efficient depletion of PIP5K1A was confirmed by western blotting (Figure 3C).

Embryos with reduced PIP5K1A exhibited developmental delays and cleavage defects. Cleavage was significantly delayed by day 2 post-activation, accompanied by abnormal division and fragmentation, and most embryos failed to progress beyond the morula stage (Figure 3D-G).

To further determine the stage-specific requirement of PIP5K1A, knockdown was performed at the germinal vesicle (GV) stage. PIP5K1A knockdown did not affect porcine oocyte maturation (Figure S4A), but significantly reduced the first cleavage rate following activation (Figure 3H, I). Notably, many oocytes remained arrested at the MII stage, indicating failed activation (Figure S4B, C). However, the distribution of PIP2 and PI4P did not change at the membrane after PIP5K1A knockdown at the 1-cell stage and cultured for 2 days (Figure S4D, E).

These results demonstrated that PIP5K1A was essential for proper oocyte activation, PIP2 synthesis, and the initiation of early embryonic cleavage, whereas PIP5K1B was dispensable in this context.

### PIP5K1A knockdown disrupts PIP2-actin co-localization and alters membrane trafficking dynamics

**Figure 4:**
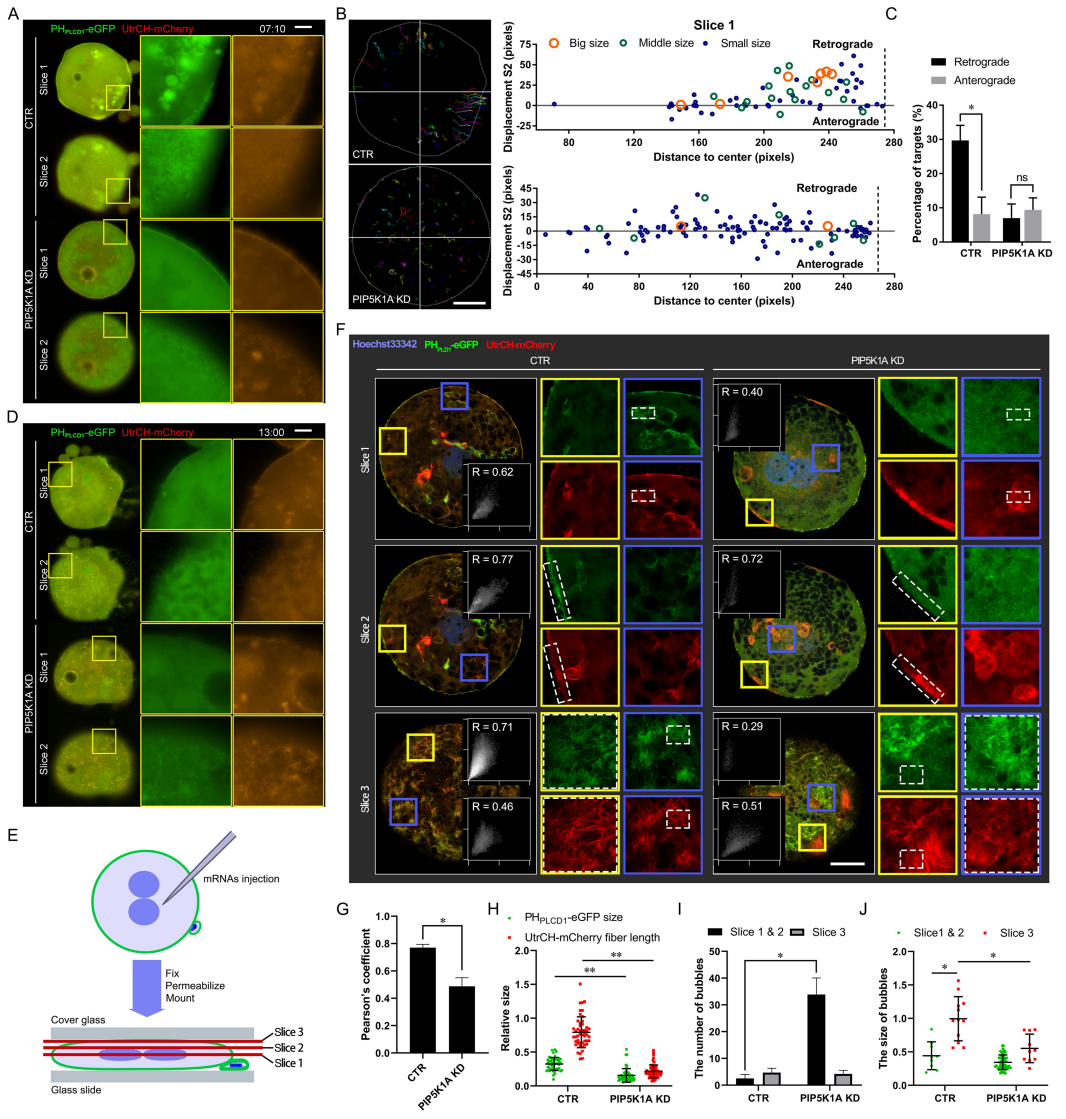
Effects of *PIP5K1A* knockdown on membrane dynamics and molecular signaling.** (A)** Live images of embryos or oocytes 7 min and 10 s after PIP5K1A knockdown in MII oocytes for 12 h and oocyte activation. Scale bar = 25 μm.** (B)** Targeting and Tracking of dots and bubbles with PH_PLCD1_-eGFP and UtrCH-mCherry in embryos or oocytes after PIP5K1A knockdown in MII oocytes for 12 hours and oocyte activation. Each track is shown in a different color, in which the lines and dots are the moving line and position in each frame, respectively. Darker lines and dots indicate later paths. Gray solid lines, shapes of embryos. Scale bar = 25 μm. The graphs show the S2 of targets of different sizes at different original positions from the center. Dotted line: Plasma membrane position.** (C)** Percentages of retrograde and anterograde targets (Fig. 4B).** (D)** Living images of activation-successful embryos 13 h after oocyte activation and PIP5K1A knockdown. Scale bar = 25 μm.** (E)** Schematic diagram showing oocytes or embryos flattened for confocal microscopy. Images of the central layer (slice 1, cytoplasm), middle layer (slice 2, cortex), and top layer (slice 3, plasma membrane) were obtained for analysis.** (F)** Confocal image of activation-successful embryos 13 h after oocyte activation and PIP5K1A knockdown. Graphs show the colocalization of PH_PLCD1_-eGFP and UtrCH-mCherry in the dotted frame. R, Pearson's coefficient. Scale bar = 25 μm.** (G)** Quantitative Pearson's coefficient in the dotted frame of slice 3 in Fig. 4F.** (H)** Relative size of PH_PLCD1_-eGFP and length of UtrCH-mCherry in slice 3.** (I)** Number of bubbles in each slice.** (J)** Size of bubbles in each slice. “*” showed a significant difference.

Live imaging was performed to assess the impact of PIP5K1A depletion on membrane dynamics. Compared with controls, knockdown embryos showed reduced PIP2- and F-actin-associated signals at the plasma membrane, accompanied by altered dynamics (Movie S2A, Figure 4A). Tracking analyses of PH_PLCD1_-eGFP and UtrCH-mCherry revealed decreased outward movement and increased inward transport (Figure 4B, C), consistent with impaired membrane trafficking.

As development progressed, vesicle-like signals partially recovered in knockdown embryos, resembling control patterns at later stages (Movie S2B, Figure 4D). To visualize subcellular localization in more detail, embryos were flattened and imaged by confocal microscopy across three optical sections: central (cytoplasmic), middle (cortical), and top (plasma membrane) layers (Figure 4E). In the apical slice, Pearson's correlation coefficient between PH_PLCD1_-eGFP and UtrCH-mCherry was significantly reduced upon PIP5K1A depletion, indicating disrupted spatial co-distribution of PIP2 and F-actin (Figure 4F, G).

Consistently, quantitative analysis demonstrated changes in PIP2 aggregate size and actin fiber length following PIP5K1A knockdown (Figure 4H). Furthermore, vesicle analysis across optical sections revealed that the knockdown group exhibited a greater number of smaller vesicles at the plasma membrane layer (Figure 4I, J), consistent with aberrant vesicle formation or trafficking.

Collectively, these results indicated that PIP5K1A was required for proper organization of PIP2-actin co-structures and directional membrane trafficking. Its loss may have contributed to signaling defects and impaired cleavage progression in early embryos.

### PIP5K1A overexpression induces excessive PIP2 vesicle formation and disrupts membrane contractility

**Figure 5:**
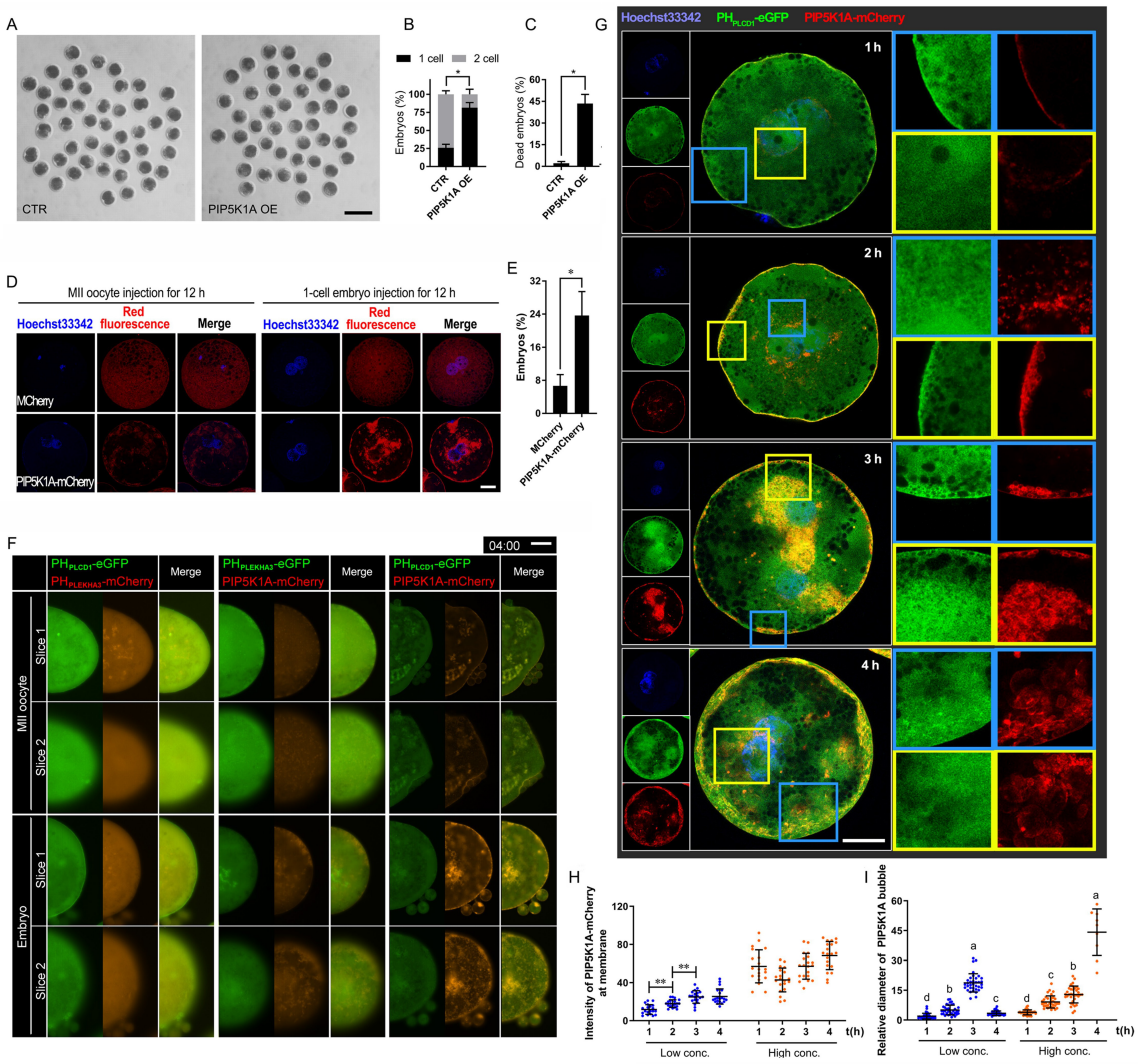
Effects of *PIP5K1A* overexpression on embryonic development and membranes.** (A)** Porcine embryonic development on day 2. Scale bar = 200 μm.** (B)** Ratio of cleaved embryos at different stages on day 2.** (C)** The ratio of dead embryos on day 2.** (D)** Specific localization of PIP5K1A-mCherry in MII oocytes and 1-cell embryos. Scale bar = 25 μm.** (E)** Ratio of activated oocytes (defined by pronuclear formation after Hoechst 33342 staining) 12 h after *PIP5K1A* overexpression without electrical stimulation for parthenogenetic activation.** (F)** Live images of PH_PLEKHA3_, PH_PLCD1,_ and PIP5K1A in MII oocytes and 1-cell embryos 4 h after their exogenous expression. Scale bar = 25 μm.** (G)** Subcellular localization of PH_PLCD1_-eGFP and UtrCH-mCherry after *PIP5K1A* overexpression. The white dotted line indicates bubbles. Scale bar = 25 μm.** (H)** The relative intensity of PIP5K1A-mCherry on the membrane at low and high concentrations of PIP5K1A-mCherry.** (I)** Relative diameters of the bubbles with PIP2-PIP5K1A at low and high concentrations of PIP5K1A-mCherry.

To assess the effects of PIP5K1A overexpression, PIP5K1A-mCherry mRNA was microinjected into MII oocytes or 1-cell embryos. Overexpression significantly increased the rates of 1-cell arrest and embryonic death compared with controls (Figure 5A-C). Notably, a subset of oocytes underwent spontaneous activation in the absence of electrical stimulation, but these embryos remained arrested at the 1-cell stage and failed to develop further (Figure 5D, E).

PIP5K1A-OE also produced bubbles, especially when injected into 1-cell embryos. The bubbles were distributed along the plasma membrane and around the cell nuclei (Figure 5D). PIP5K1A-mCherry and PH_PLCD1_-eGFP in embryos colocalized in all membranes except the nucleus and formed clustered plaques in oocytes and bubbles in embryos near the plasma membrane and in the deep cytoplasm near the nucleus (Figure 5F, Figure S5A).

Live imaging revealed dose-dependent changes in membrane dynamics. At early time points, PIP5K1A and PIP2 were primarily localized at the plasma membrane, whereas increased expression led to the formation and growth of PIP2-enriched vesicles near the membrane and perinuclear regions (Figure S5B; Figure 5G-I; Movie S3A). Once the bubbles moved to the deep cytoplasm, the PIP2-insufficient plasma membrane expanded outward, and the embryos eventually died (Movie S3B). Simultaneously, PIP5K1A-mCherry did not colocalize with PH_PLEKHA3_-eGFP, but PIP5K1A-mCherry-positive bubbles were distributed around the dense area of PH_PLEKHA3_-eGFP (Movie S3C). As time progressed, larger bubbles appeared in the outer layer. However, PIP5K1A-mCherry gradually colocalized with PH_PLEKHA3_-eGFP near the plasma membrane in MII oocytes (Figure S5A, B).

**Figure 6:**
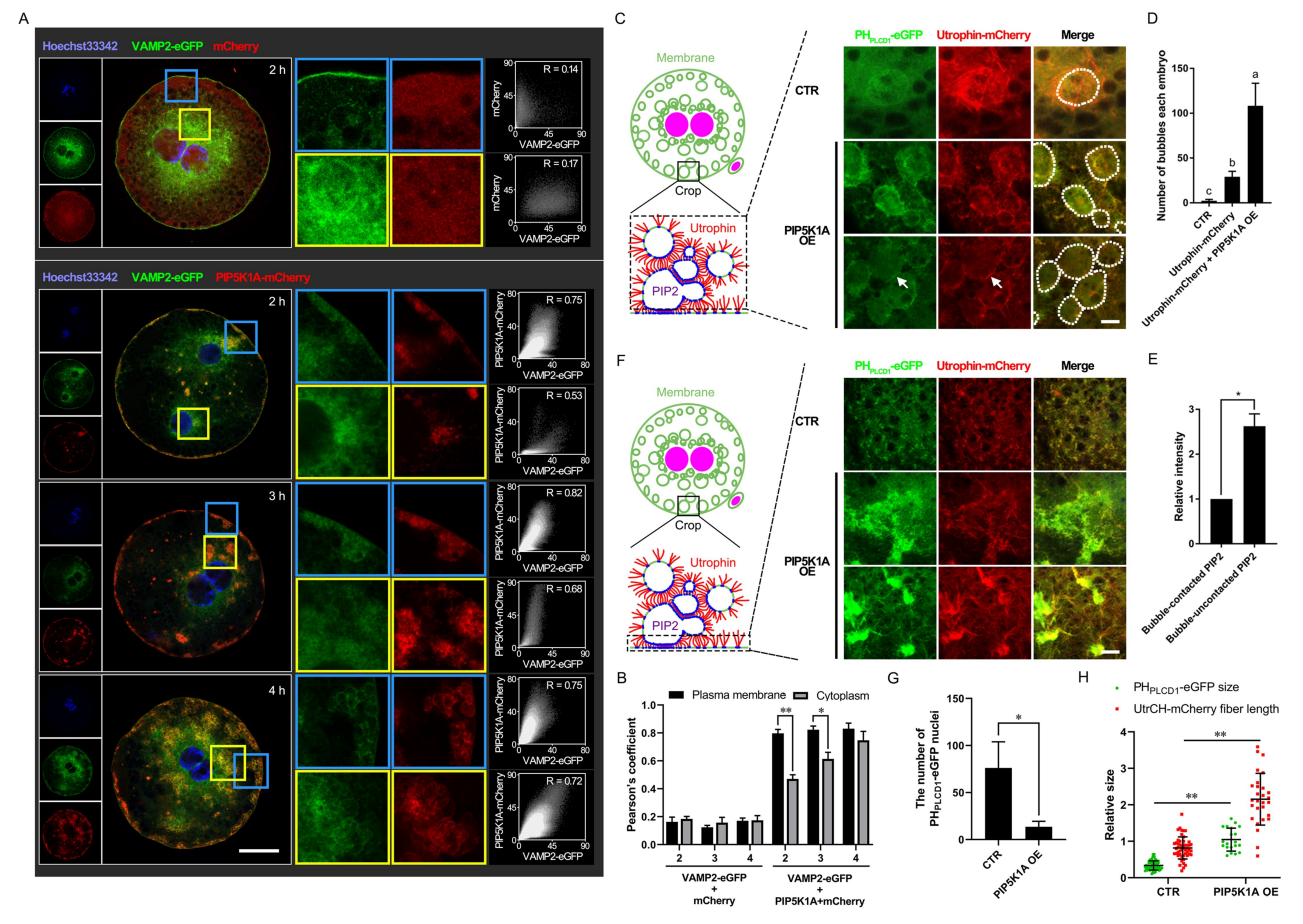
Effects of *PIP5K1A* overexpression on membranous dynamics and its related cytoskeleton.** (A)** Localization of VAMP2-eGFP and PIP5K1A-mCherry. Scale bar = 25 μm.** (B)** Quantitative Pearson's coefficient in Fig. 6A.** (C)** Subcellular localization of PH_PLCD1_-eGFP and UtrCH-mCherry after *PIP5K1A* overexpression. The white dotted circles indicate the bubbles. The arrows indicate membrane-contacted bubbles. Scale bar = 10 μm.** (D)** Number of bubbles in each embryo when PIP5K1A was overexpressed.** (E)** The relative intensity of PH_PLCD1_-eGFP in different bubble regions.** (F)** Subcellular localization of PH_PLCD1_-eGFP and UtrCH-mCherry at or near the plasma membrane after *PIP5K1A* overexpression. Scale bar = 10 μm.** (G)** Number of PH_PLCD1_-eGFP nuclei in Fig. 6E when PIP5K1A was overexpressed.** (H)** Size of PH_PLCD1_-eGFP and fiber length of UtrCH-mCherry (Fig. 6E) when PIP5K1A was overexpressed.

Further analysis showed that vesicle-associated proteins and cytoskeletal components were affected. VAMP2 initially localized to the plasma membrane together with PIP5K1A, and later accumulated in cytoplasmic vesicles (Figure 6A, B). F-actin structures were reorganized, with vesicles moving along actin fibers and forming larger aggregates (Figure 6C-F). This resulted in fewer PH_PLCD1_-eGFP nuclei and longer F-actin fibers in the PIP5K1A OE group (Figure 6G, H). In summary, PIP5K1A OE led to excessive PIP2 production and vesicle formation, which in turn sequestered F-actin and disrupted plasma membrane contractility.

### PIP5K1A regulates RhoA activity and modulates the PLC-IP3-Ca^2+^ signaling axis

**Figure 7:**
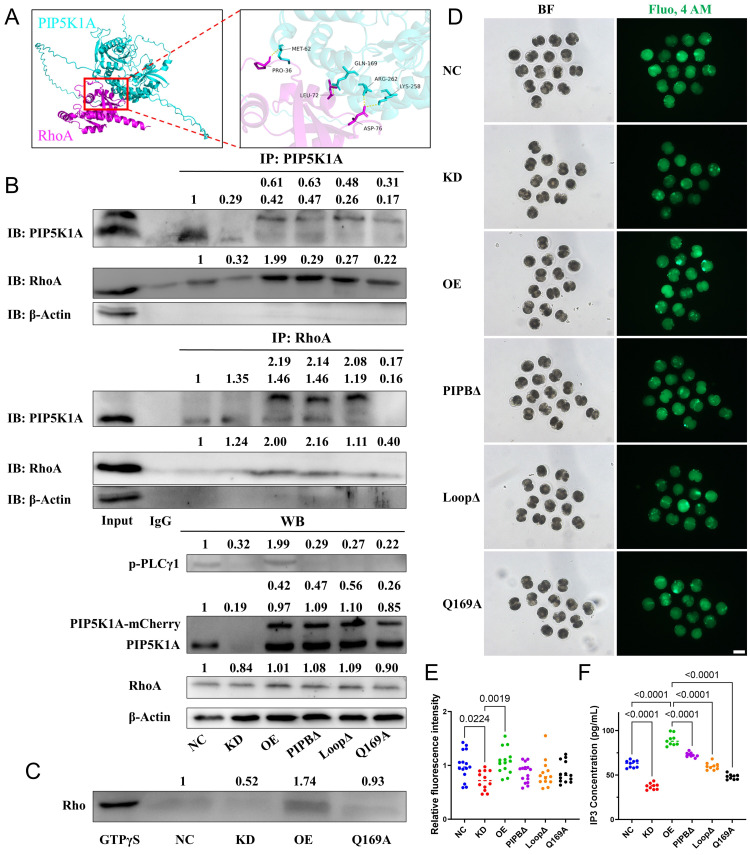
PIP5K1A-RhoA interaction supports PLCγ1 activation, IP3 production, and Ca^2+^ signaling in porcine embryos.** (A)** Structural model of the PIP5K1A-RhoA complex, highlighting key contact residues.** (B)** Co-immunoprecipitation showing reciprocal association of PIP5K1A and RhoA in control (NC), PIP5K1A knockdown (KD), overexpression (OE), and mutant embryos (PIPBΔ, LoopΔ, Q169A). Lower blots show phosphorylated PLCγ1 (p-PLCγ1), PIP5K1A-mCherry, endogenous PIP5K1A, RhoA, and β-actin. The numbers above the bands represent their relative grayscale values.** (C)** Pull-down assay measurement of active RhoA-GTP levels.** (D)** Representative bright-field (BF) and Fluo-4 AM images indicating intracellular Ca^2+^ signals. Scale bar = 100 μm.** (E)** Quantification of Fluo-4 AM fluorescence intensity.** (F)** IP3 concentration measured by ELISA.

To examine the role of PLC-mediated PIP2 turnover, PLCD1 (PLCδ1) was perturbed. PLCD1 knockdown impaired oocyte activation and delayed early cleavage, phenocopying key aspects of PIP5K1A depletion (Figure S6). In parallel, phosphorylated PLCγ1 (p-PLCγ1), used as a readout of PLC pathway activity, was reduced upon PIP5K1A knockdown and increased with overexpression (Figure 7B). Consistently, IP3 levels showed similar changes (Figure 7F), indicating altered PIP2-dependent signaling.

In a Ca^2+^-free medium, PIP2-enriched bubbles gathered near the plasma membrane with increased intensity of PH_PLCD1_-eGFP. When CB reduced actin fibers, the distribution of bubbles became random, and their movement decreased (Figure S8). Intracellular Ca^2+^ levels showed corresponding changes (Figure 7D, E), supporting an association between PIP5K1A activity and PLC-Ca^2+^ signaling.

We next examined RhoA-related signaling. Knockdown of PIP5K1A did not affect the total protein levels of anillin (Figure S7B, C), RhoA, or ROCK1 (Figure S7D, E) compared to controls. However, membrane-localized RhoA (P1) was reduced, while cytoplasmic RhoA (S) increased in the PIP5K1A knockdown group (Figure S7F, G). Notably, anillin, a known membrane marker during cytokinesis, was exclusively localized to the membrane (P1, P2; Figure S7F, G).

The structural prediction and Co-immunoprecipitation suggested a potential interaction between PIP5K1A and RhoA (Figure 7A, B). In addition, GTP pull-down assays showed that active RhoA levels decreased upon knockdown and increased with overexpression (Figure 7C).

Together, PIP5K1A promoted RhoA activation and sustained PLC-IP3-Ca^2+^ signaling by maintaining PIP2 levels at the membrane, thereby supporting membrane remodeling.

### The PIPB motif and activation loop of PIP5K1A are essential for lipid binding, vesicle formation, and RhoA signaling

Domain-specific functions of PIP5K1A were dissected through the construction and analysis of several lipid-binding mutants. The catalytically inactive mutant PIP5K1A^Q169A^, which retains membrane targeting via the disheveled-binding site but lacks enzymatic conversion of PI4P to PIP2 31, impaired cytokinesis and reduced RhoA and ROCK1 protein levels (Figure S7A, D, E). Additionally, Q169 was one of the binding sites for RhoA and PIP5K1A (Figure 7A). The Q169A mutation led to reduced binding affinity with PIP5K1A (Figure 7B). Furthermore, Q169 was critical for maintaining RhoA activity (Figure 7C) and reduced intracellular Ca^2+^ concentration and IP3 levels (Figure 7D-F).

**Figure 8:**
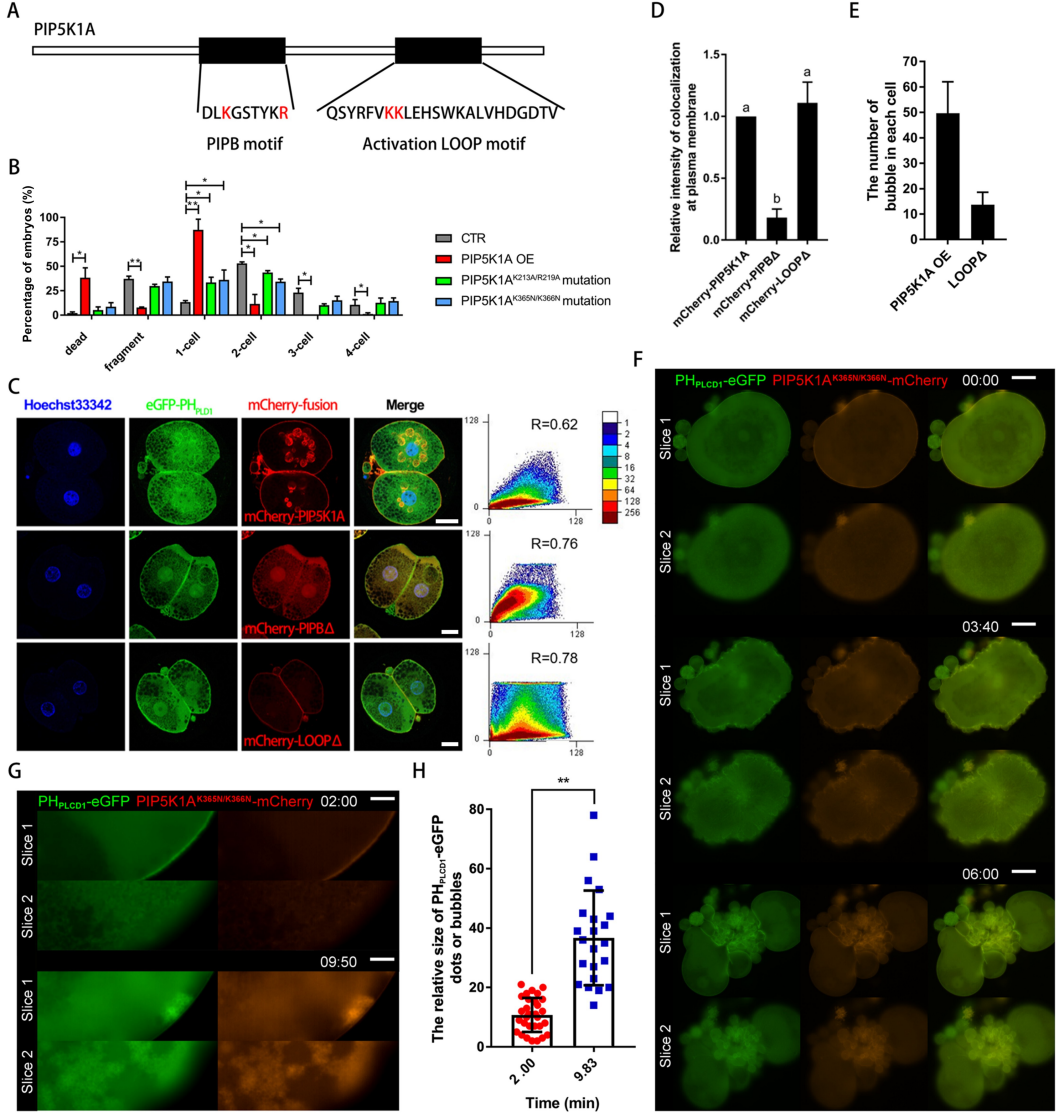
Function of phosphatidylinositol-binding domains in PIP5K1A.** (A)** Schematic diagram showing the sequences of the PIPB motif and the activation loop motif. Red letters indicate key amino acid residues that bind phosphatidylinositol.** (B)** Percentage of embryos at different stages on day 2 after PIPB motif or activation loop motif mutation.** (C)** Subcellular co-localization of PH_PLCD1_-eGFP with PIP5K1A-mCherry, PIP5K1A^K213A/R219A^ mCherry, or PIP5K1A^K365N/K366N^-mCherry mutants. R, Pearson's coefficient. Scale bar = 25 μm.** (D)** The relative intensity of colocalization at the plasma membrane or bubbles between PH_PLCD1_-eGFP and PIP5K1A-mCherry, PIP5K1A^K213A/R219A^-mCherry mutant, and PIP5K1A^K365N/K366N^-mCherry in Fig. 8C,** (E)** Number of bubbles in blastomeres of embryos in the PIP5K1A-mCherry overexpression group and PIP5K1AK^365N/K366N^-mCherry mutant group.** (F)** Live imaging of PH_PLCD1_-eGFP and PIP5K1A^K365N/K366N^-mCherry cells during cytokinesis. Scale bar = 25 μm.** (G)** Membrane aggregation, bubble formation, and transfer into the cytoplasm of PH_PLCD1_-eGFP and PIP5K1A^K365N/K366N^-mCherry. Scale bar = 25 μm.** (H)** The evenness index of PH_PLCD1_-eGFP at the plasma membrane (Fig. 7G) and the size of the bubbles under the plasma membrane.

Two additional regions were examined, including the PIPB motif (K213A/R219A) and the activation loop (K365N/K366N) (Figure 8A). Unlike wild-type overexpression, which caused embryonic lethality, both mutants led to developmental arrest prior to the blastocyst stage (Figure 8B). The PIPB mutant presented in the cytoplasm and nucleus but did not colocalize with PIP2 signal outside the nucleus and formed bubbles. In contrast, the activation loop mutant formed fewer vesicle-like structures and displayed altered cortical dynamics during cell division (Figure 8C-H; Movie S4). During cytokinesis, the aggregated PIP5K1A^K365N/K366N^-mCherry nucleus at the membrane had more powerful contractility than its surrounding area and was trapped in the cytoplasm, and the growth of these fibers at the outer surface of the plasma membrane was greatly promoted (Figure 8F; Movie S4A, B). Excess expression of PIP5K1A^K365N/K366N^-mCherry over a long time resulted in increased nuclear aggregation at the plasma membrane. These nuclei developed from dots into bubbles, aggregated, and moved into the cytoplasm at the 1-cell stage (Figure 8G, H; Movie S4C). These dynamic phenomena were particularly evident at one position in the plasma membrane, which may have determined the cell's polarity (Movie S4D).

Live imaging further revealed that the activation loop mutant exhibited abnormal redistribution between the plasma membrane and cytoplasm during cytokinesis, with inconsistent recruitment to the cleavage furrow (Figure 8F, Movie S5).

Collectively, these findings indicated that the PIPB motif anchored PIP5K1A to the membrane-bound PIP2, which maintained cortical morphology and supported spindle capture during cytokinesis. In contrast, the activation loop was essential for catalytic activity and RhoA-dependent endocytosis. Both domains were required for efficient PIP2 production and proper signal transduction in early embryonic development.

## Discussion

Phosphoinositide metabolism generates distinct lipid species with spatiotemporal specificity, supporting diverse cellular processes during early embryogenesis 32. In mammalian embryos, PTEN, PIP5K1, and PIP4K2 family members contribute to the production of phosphatidylinositol 4,5-bisphosphate (PIP2) at different subcellular locations. In mouse oocytes, PIP2 has been implicated in cortical actin organization, spindle positioning 33, and Ca^2+^ oscillations -dependent activation through PLC-mediated hydrolysis 34, 35. While previous studies in Drosophila embryos have highlighted dynamic PIP2 redistribution during development 36, mouse embryos undergo cellular cleavage and display dynamic PIP2 redistribution associated with membrane remodeling and cytokinesis 37. Its spatial regulation and functional contribution in large mammalian embryos remain incompletely understood.

In this study, we identify a spatially partitioned mechanism of PIP2 production in porcine embryos. Our data support that PTEN-associated PIP2 is primarily localized to nuclear and spindle regions, consistent with previous findings 12, 13. whereas PIP5K1A contributes to membrane-associated PIP2 derived from PI4P. Notably, PIP5K1A sustains basal PIP2 levels throughout preimplantation development, differing from the transient enrichment patterns reported in other species 6.

Functionally, knockdown of PIP5K1A did not overtly disrupt the spatial localization of PIP2 but significantly delayed the formation of cortical PIP2-F-actin clusters, suggesting that PIP5K1A regulates the kinetics rather than the positioning of PIP2 structures at the membrane. In addition, despite high sequence similarity 31, PIP5K1A and PIP5K1B exhibited non-redundant roles, consistent with context-dependent functional divergence reported in other systems 20, 21.

Consistent with the phenotypic similarity between PIP5K1A and PLCD1 depletion, both proteins were found to colocalize with PIP2-enriched membrane regions, supporting a spatial association between PIP2 synthesis and hydrolysis 7, 21. These observations suggest that PIP2 may act as a dynamically regulated intermediate maintained by the balance between PIP5K1A activity and PLC-mediated turnover. This dynamic pool of PIP2 is tightly regulated by Ca^2+^ signaling, which activates PLC and triggers PIP2 turnover 38. In oocytes, intracellular Ca^2+^ increases following activation, which is essential for the enhancement of membrane dynamics 39-41. PIP5K1 activity has been shown to elevate cytoplasmic Ca^2+^ levels by promoting IP3 production 3, 21. Our data, together with previous studies, are consistent with a model in which PIP5K1A-dependent PIP2 availability contributes to sustained PLC-IP3-Ca^2+^ signaling, potentially forming a feedback loop that supports membrane remodeling. At the plasma membrane, PIP2-enriched nanodomains on the inner leaflet serve as nucleation hubs for cortical F-actin assembly by recruiting scaffold proteins such as Actin-related protein 2/Actin-related protein 3 complex 42, 43. Consistent with this, F-actin, each monomer of which is marked by the CH1 domain of utrophin 5, was observed radially arrayed around PIP2 foci in a pattern resembling astral structures. These PIP2-F-actin clusters support contractile membrane domains, serving as anchors for mechanical tension. Overexpression of the PIP5K1A^K365N/K366N^ mutant—impaired in catalytic activity but still membrane-associated—induced cortical actin aggregation, supporting a role for PIP5K1A as an upstream nucleation signal. Live imaging also revealed filamentous structures extending along the outer membrane surface, which represent cortical cytoskeletal networks linked to PIP2-rich domains. The enhanced contractility of these regions suggests that PIP2 functions not only as a signaling lipid but also as a mechanical anchor that organizes membrane-cytoskeleton coupling. Interestingly, reduced colocalization between PH_PLCD1_-eGFP and UtrCH-mCherry was observed during cytokinesis, the basis of which remains unclear. This may reflect localized turnover of PIP2, spatial rearrangement of F-actin, or regulatory uncoupling under high contractile stress.

As development progresses, overexpressed PIP5K1A drives PIP2 accumulation at the plasma membrane, followed by vesicle formation and internalization of PIP2-enriched domains into the cytoplasm. This PIP2 internalization is accompanied by reduced membrane availability of PIP2 for PLC-mediated hydrolysis. The resulting imbalance contributes to defects in membrane turnover and contractility observed in the PIP5K1A knockdown group. Despite their antagonistic enzymatic roles, PIP5K1A and PLC may function cooperatively, forming a Ca^2+^-sensitive module to regulate dynamic PIP2 homeostasis, as reported previously 7. Once the dynamics were disrupted—as in knockdown embryos—membrane-bound F-actin accumulates, further impairing membrane remodeling. The ability of PIP5K1A overexpression to promote PIP2 internalization is consistent with previous reports on PIP5K1B-mediated endocytosis 6, likely due to conserved structural features between these isoforms.

Importantly, these dynamics were characterized in a PA model and should therefore be interpreted as a cell-autonomous maternal program of PIP2-dependent membrane remodeling 44. Because PA lacks paternal contributions present in bi-parental fertilization—including sperm-derived PLCζ and centrioles—the timing, symmetry, and kinetics of downstream membrane and cleavage dynamics may differ in fertilized embryos.

PIP2 has been widely implicated in both exocytic and endocytic membrane trafficking, acting through Ca^2+^-dependent vesicle fusion 45 and clathrin- or small GTPase-associated internalization pathways 3, 46. These opposing yet coupled functions underscore the role of PIP2 as a central coordinator of membrane trafficking 4. Our findings support this dual role. Before oocyte activation, endogenous PIP5K1A remains cytoplasmic and catalytically inactive. Upon activation, PIP5K1A-mCherry relocalizes to the cortex, where it converts PI4P to PIP2 and colocalizes with VAMP2, a v-SNARE protein, at vesicular structures 14. This spatial and temporal shift coincides with membrane fusion events, implicating PIP2 synthesis in exocytosis initiation.

In addition to membrane trafficking, PIP5K1A may be associated with cytoskeletal regulation through small GTPase pathways. In particular, perturbation of the activation loop altered vesicle formation and membrane dynamics, consistent with disrupted endocytic processes. While previous studies have implicated Rac1-dependent mechanisms in PIP2-mediated membrane remodeling 8, our data support a potential association between PIP5K1A activity and these pathways.

RhoA is known to accumulate at the equatorial cortex in the central spindle during anaphase 10, promoting localization of the cleavage furrow at the membrane and cytokinesis initiation 18, 47. In addition, the production of a new plasma membrane during cytokinesis requires anterograde PI4P-positive vesicles 48, and active RhoA-GTP at the membrane dynamically colocalizes with PIP2 13. PIP5K1A knockdown disrupted membrane-bound RhoA accumulation, supporting the notion that PIP2 serves as a spatial anchor for RhoA. Overexpression of the catalytically inactive K365N/K366N mutant restored PIP2 clustering and partially recruited RhoA to the furrow and spindle midzone, suggesting that membrane-localized PIP2, rather than catalytic activity alone, is sufficient for RhoA anchoring.

Although small GTPases such as Rac1 and RhoA have been implicated in distinct aspects of membrane dynamics, the precise relationship between these pathways in early embryos remains to be fully defined. The abnormal cleavage phenotypes observed in some embryos further suggest that disruption of membrane-associated signaling may uncouple cytokinetic progression from cytoskeletal organization 11.

Consistent with its role in PIP2 production, PIP5K1A activity was closely linked to PLC enzymes 49 and is essential for IP3 generation and subsequent Ca^2+^ release 50, 51. Both PIP5K1A knockdown and the Q169A mutant resulted in reduced IP3 levels and attenuated cytosolic Ca^2+^ responses, supporting a requirement for PIP5K1A-dependent PIP2 availability in sustaining this signaling axis. The PIPB motif emerges as a key determinant for stable membrane association of PIP5K1A. Mutation of this region (K213A/R219A) altered subcellular localization and weakened recruitment of signaling components such as RhoA and p-PLCγ1, potentially disrupting the spatial coordination of cytokinesis and membrane remodeling.

Both the PIPB motif and the activation loop domains of PIP5K1A can bind to PI4P- or PIP2-membranes, and mutations in these regions produced distinct phenotypic effects in embryos. The head of PIP2 is rich in negative charges owing to its phosphate groups. The protein sequence of PIP5K1A near the PIPB motif contained dense basic amino acid residues that formed a rigid contact surface 31. The activation loop is a flexibly stretched structure that contains basic amino acid residues 31. This activation loop is essential for PI4P processing in the membrane 9. These characteristics support our findings that PI4P is only present in the PIPB motif via the activation loop and that PIP2 synthesized from PI4P remains stable in combination with the PIPB motif. In addition, both domains are essential for the binding of PI4P to PIP5K1A. In addition, it is worth noting that the PIPB motif mutant is localized in the nucleus because of the presence of nuclear PIP2. PIP5K1A may have both nuclear and cytoplasmic localization.

Although these domain-specific perturbations revealed clear phenotypic associations with cortical organization and RhoA-related membrane dynamics, we did not directly measure cortical tension or endocytic flux in this study. Therefore, our interpretations are based primarily on localization and phenotypic analyses. Future studies incorporating quantitative measurements of membrane mechanics and trafficking will help clarify the mechanistic roles of these domains.

Phosphoinositide dysregulation has been associated with defects in membrane trafficking and cytokinesis in mammalian systems 52, 53. Excessive PIP2 accumulation may alter membrane curvature 54, actin polymerization dynamics 20, and vesicle trafficking efficiency 55. Consistent with this, our dose-dependent experiments indicate that precise regulation of PIP5K1A activity is required to maintain membrane lipid balance during early cleavage. Moderate overexpression increased membrane-associated PIP2 without severe developmental defects, whereas higher expression levels led to excessive vesicle formation and membrane aggregation. These observations suggest that the most severe phenotypes likely reflect supraphysiological PIP2 accumulation rather than physiological regulation, and therefore overexpression should be interpreted as a dosage-sensitive perturbation model.

PIP5K1A may function in feedback, as reported previously 21, which was consistent with the bubbles induced by PIP5K1A-mCherry, UtrCH-mCherry, or VAMP2-eGFP overexpression and Ca^2+^-induced oocyte activation in this study. Given the different distribution and size of bubbles and different colocalization, F-actin promotes nucleation; VAMP2 induces membrane fusion; PIP5K1A tethers vesicles to the plasma membrane, provides and aggregates PIP2, and promotes endocytosis with Rac1 or cytokinesis with RhoA; and Ca^2+^ induces the activation of PLC for PIP2 hydrolysis and membrane fusion. Some proteins interact with PIP5K1A and function via different feedback mechanisms. For example, lysophospholipids and arachidonic acid derived from PIP2 by PLC and PLA, respectively, promote membranes 56.

**Figure 9:**
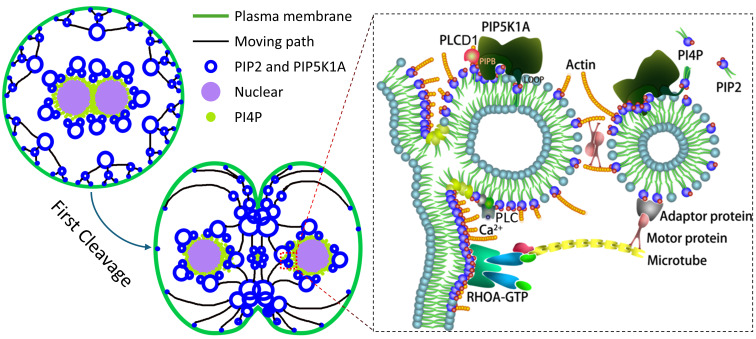
** Proposed model showing how PIP5K1A regulates membrane dynamics during oocyte activation and early embryonic cleavage.** Following activation, PI4P-containing vesicles are transported to the cortex, where PIP5K1A promotes local PIP2 enrichment and membrane remodeling. PIP5K1A-associated PIP2 domains facilitate vesicle fusion, actin organization, and membrane contractility in a Ca2+- and cytoskeleton-dependent manner. During cytokinesis, PIP2-enriched membrane regions promote RhoA recruitment and contractile ring assembly to coordinate cleavage-associated membrane remodeling.

In summary, our findings support a working model in which PIP5K1A orchestrates a spatially and temporally regulated phosphoinositide signaling cycle that drives membrane remodeling during early embryogenesis (Fig. 9). PI4P-containing vesicles are delivered toward the cortex and, following activation, PIP5K1A relocalizes to membrane-proximal structures where it promotes PIP2 enrichment (Fig. 2, Fig. 6). In this model, PIP5K1A-mediated PIP2 production and membrane association are consistent with a role in organizing vesicle fusion and cortical F-actin assembly (Fig. 5-8). Subsequent Ca^2+^-dependent PLC activity and downstream IP3/Ca^2+^ signaling are supported by changes in PLCγ1 phosphorylation and IP3/Ca^2+^ readouts upon PIP5K1A perturbation, which is consistent with hydrolysis-driven turnover of PIP2-enriched structures. During cytokinesis, PIP2-enriched membrane domains may act as hubs for RhoA recruitment and contractile ring assembly, in line with reduced membrane-associated RhoA upon PIP5K1A knockdown and evidence for PIP5K1A-RhoA association (Fig. 7). Together, these observations are consistent with a self-reinforcing regulatory circuit that couples vesicle trafficking, actin remodeling, and Ca^2+^ signaling to coordinate membrane contractility during cleavage.

From a translational perspective, precise regulation of membrane dynamics, cytoskeletal integrity, and Ca^2+^ signaling is critical for embryo developmental competence. Given the physiological similarities between porcine and human embryos, modulation of PIP5K1A-mediated PIP2 dynamics may provide a potential strategy to improve *in vitro* embryo production systems and inform optimization of assisted reproductive technologies.

This study was performed using *in vitro*-cultured parthenogenetically activated embryos, which do not fully recapitulate fertilization-associated processes, including paternal contributions. In addition, although RNAi-mediated knockdown and domain perturbations produced consistent phenotypes, off-target effects and the absence of rescue experiments remain limitations. Future studies employing genetic models and integrated lipidomic and proteomic approaches will be required to further define the molecular mechanisms underlying PIP5K1A function in early embryogenesis.

## Supplementary Material

Supplementary figures and tables, movie legends.

Supplementary movie 1.

Supplementary movie 2.

Supplementary movie 3.

Supplementary movie 4.

Supplementary movie 5.

## Data Availability

The authors declare that all primary data points are displayed in the manuscript and its corresponding Supplementary Information files. All raw data supporting the findings of this study are available from the corresponding author upon request.
