# Supplementary material for: PIP5K1A-PIP2-RhoA Signaling Orchestrates Membrane Remodeling during Early Porcine Embryo Development
Source: Int J Biol Sci. 2026 May 15;22(10):5320–39. doi: 10.7150/ijbs.129307 (PMC13215358; doi:10.7150/ijbs.129307)
Supplement: Supplementary file 1 — Supplementary figures and tables, movie legends. [file ijbsv22p5320s1.pdf]

Supporting information

PIP5K1A–PIP2–RhoA Signaling Orchestrates Membrane Remodeling during Early Porcine Embryo Development

Cheng-Lin Zhan, Song-Hee Lee, Zheng-Wen Nie, Xiang-Shun Cui

Corresponding authors: Prof. Dr. Xiang-Shun Cui and Dr. Zheng-Wen Nie

Email: Xiang-Shun Cui: xscui@cbnu.ac.kr; Zheng-Wen Nie: 1079537899@qq.com

The file includes:

Figures. S1 to S8

Tables S1 and S2

Other Supplementary Materials for this manuscript include the following:

Movie S1 to S5

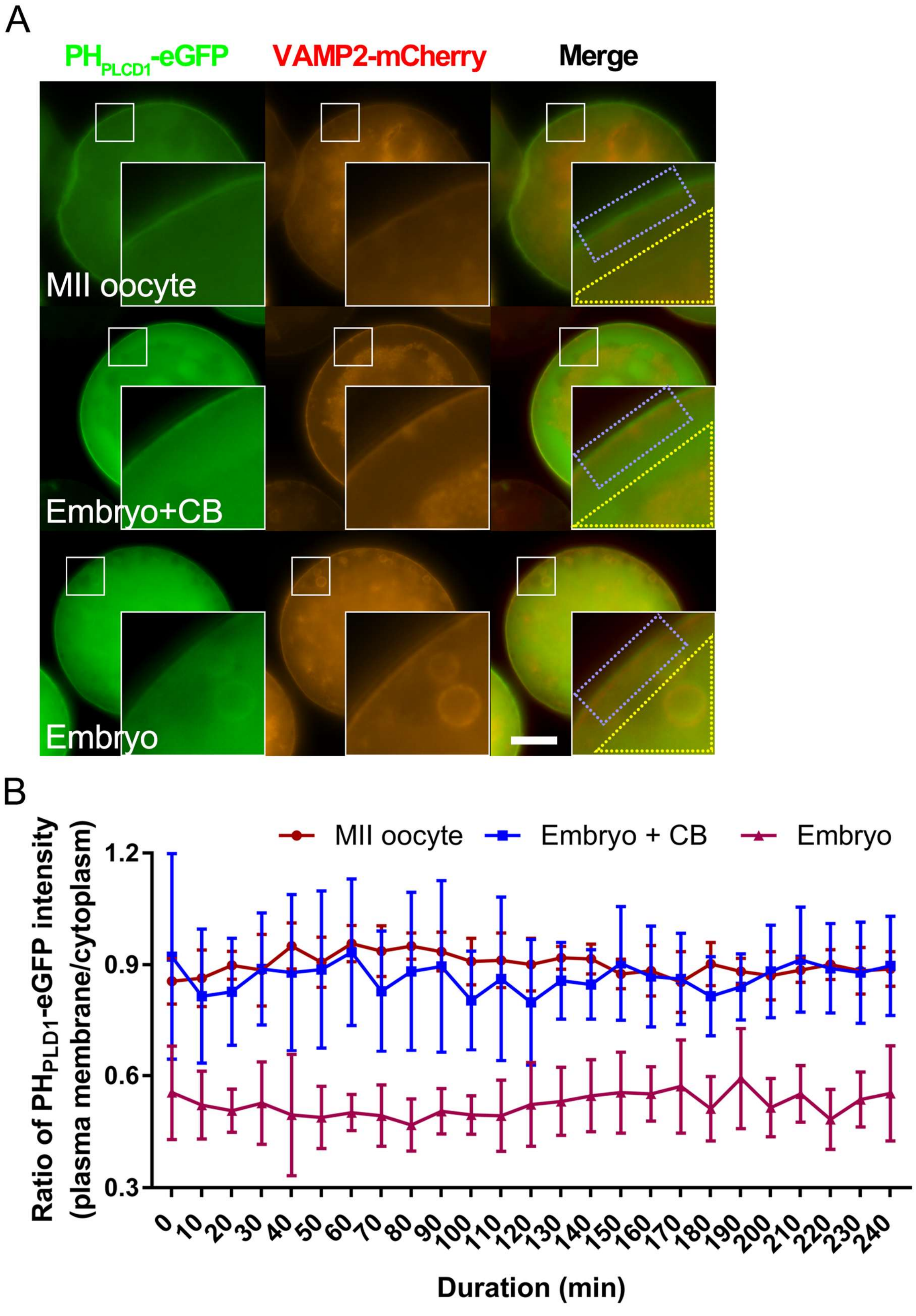
A
PH$_{PLCD1}$-eGFP
VAMP2-mCherry
Merge
MII oocyte
Embryo+CB
Embryo
B
MII oocyte
Embryo + CB
Embryo
Ratio of PH$_{PLD1}$-eGFP intensity (plasma membrane/cytoplasm)
1.2
0.9
0.6
0.3
0
10
20
30
40
50
60
70
80
90
100
110
120
130
140
150
160
170
180
190
200
210
220
230
240
Duration (min)

Figure S1. Live imaging of PH_{PLCD1}-eGFP and VAMP2-mCherry in MII oocytes and embryos. **(A)** The Distribution of PH_{PLCD1}-eGFP and VAMP2-mCherry in MII oocytes and embryos. CB, cytochalasin B. Scale bar = 25 μm. **(B)** Ratio of PH_{PLCD1}-eGFP in the cytoplasm to the plasma membrane.

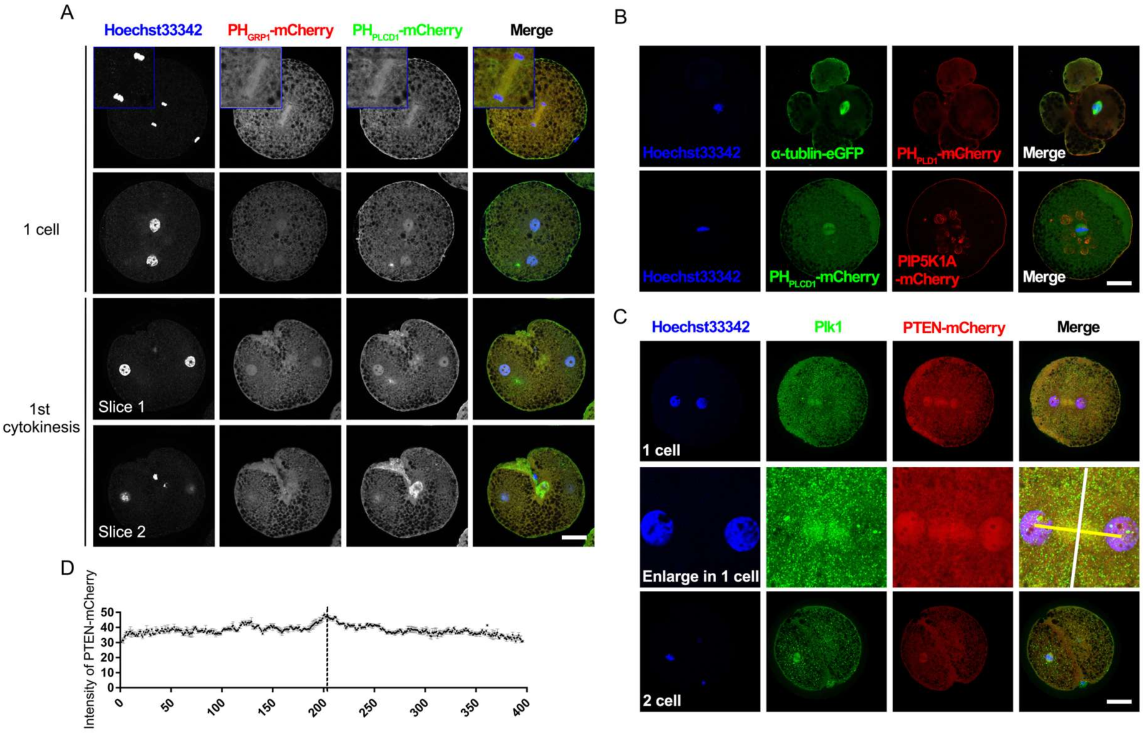

Figure S2. Localization of PIP3, PIP2, and their related proteins in the nucleus or at the spindle. **(A)** Localization of PH_{GRP1}-mCherry (a PIP3 marker) and PH_{PLCD1}-eGFP in the nucleus and spindle. **(B)** Subcellular localization of PIP2 and PIP5K1A in the spindles. **(C)** Subcellular localizations of Plk1 (spindle) and PTEN. **(D)** The intensity of PTEN-mCherry along the spindle axis (yellow line) in Figure S2C. The dotted line represents the white line in Figure S2C. Scale bar = 25 μm.

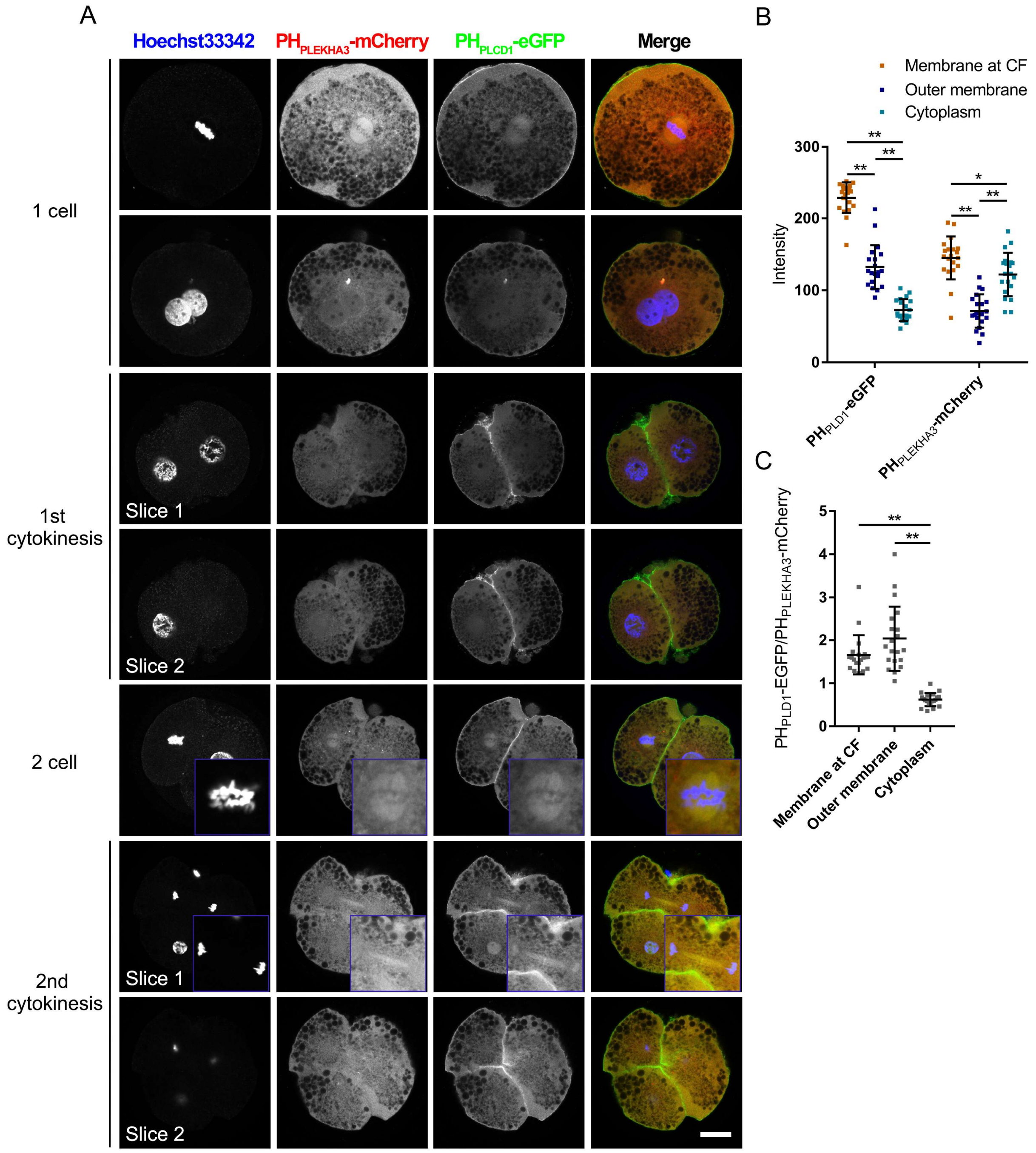

Figure S3. Localization of PI4P and PIP2 at the membrane and spindle. (A) Localization of $PH_{PLEKHA3}$-mCherry and PH_{PLCD1}-eGFP at the membrane and spindle during 1-cell, 2-cell, and cytokinesis stages. **(B)** The intensities of $PH_{PLEKHA3}$-mCherry and PH_{PLCD1}-eGFP in different

embryonic regions during cytokinesis. **(C)** Intensity ratio of PH_{PLCD1}-eGFP to $PH_{PLEKHA3}$-mCherry in different regions of the embryo during cytokinesis. Scale bar = 25 µm.

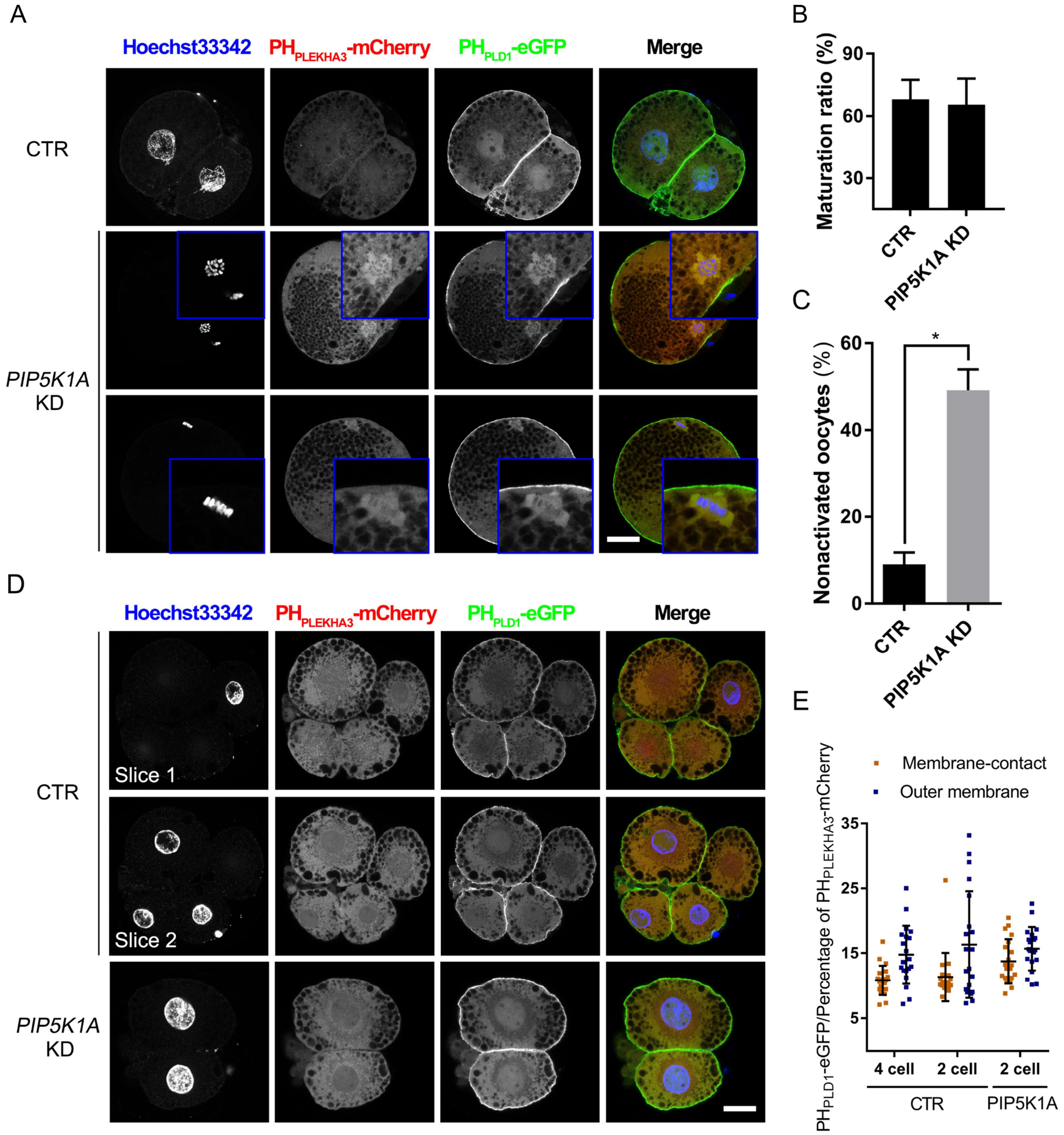

Figure S4. Effects of *PIP5K1A* knockdown in oocytes and embryos on PI4P and PIP2. (A) $PH_{PLEKHA3}$-mCherry and PH_{PLCD1}-eGFP expression 44 h after PIP5K1A knockdown in GV oocytes and 1 d after parthenogenetic electrical stimulation for oocyte activation. Scale bar = 25 μm. **(B)** Maturation ratio of GV stage oocytes at 44 h after PIP5K1A knockdown. **(C)** Percentage of non-activated GV oocytes 44 h after PIP5K1A knockdown and 1 d after parthenogenetic

electrical stimulation. **(D)** $PH_{PLEKHA3}$-mCherry and PH_{PLCD1}-eGFP 1 d after PIP5K1A knockdown of 1-cell embryos. Scale bar = 25 μm. **(E)** Intensity ratio of PH_{PLCD1}-eGFP to $PH_{PLEKHA3}$-mCherry in different regions of the embryo (Figure S4D).

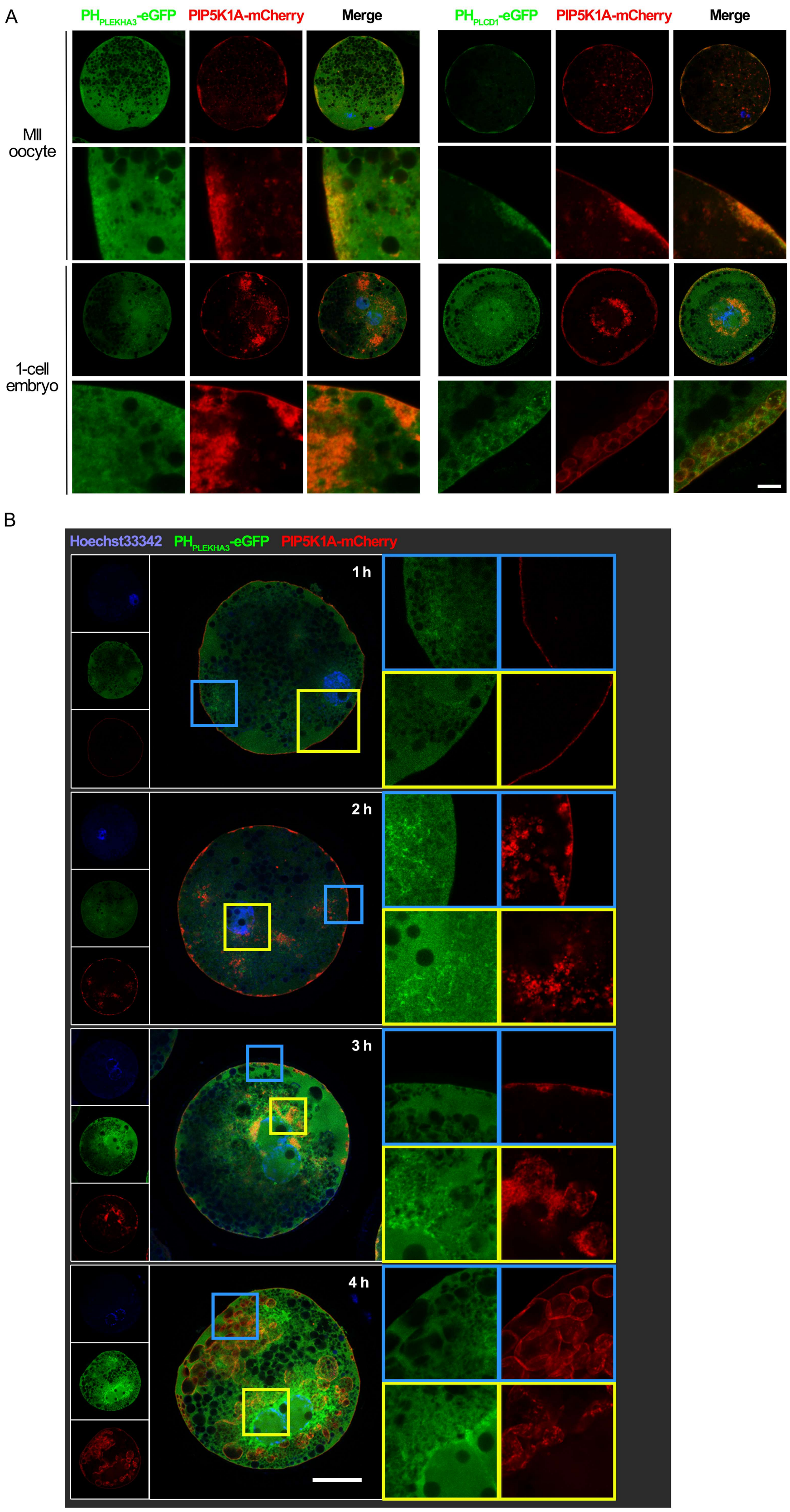
A
$PH_{PLEKHA3}$-eGFP
PIP5K1A-mCherry
Merge
PH_{PLCD1}-eGFP
PIP5K1A-mCherry
Merge
MII oocyte
1-cell embryo
B
Hoechst33342
$PH_{PLEKHA3}$-eGFP
PIP5K1A-mCherry
1 h
2 h
3 h
4 h

Figure S5. Effects of *PIP5K1A* overexpression on PIP2 and PI4P. (A) Localization of $PH_{PLEKHA3}$-mCherry, PH_{PLCD1}-eGFP, and PIP5K1A-mCherry 4 h after exogenous expression in MII oocytes. Scale bar = 25 µm. **(B)** The localization of $PH_{PLEKHA3}$-eGFP and PIP5K1A-mCherry after their exogenous expression in embryos. Scale bar = 25 µm.

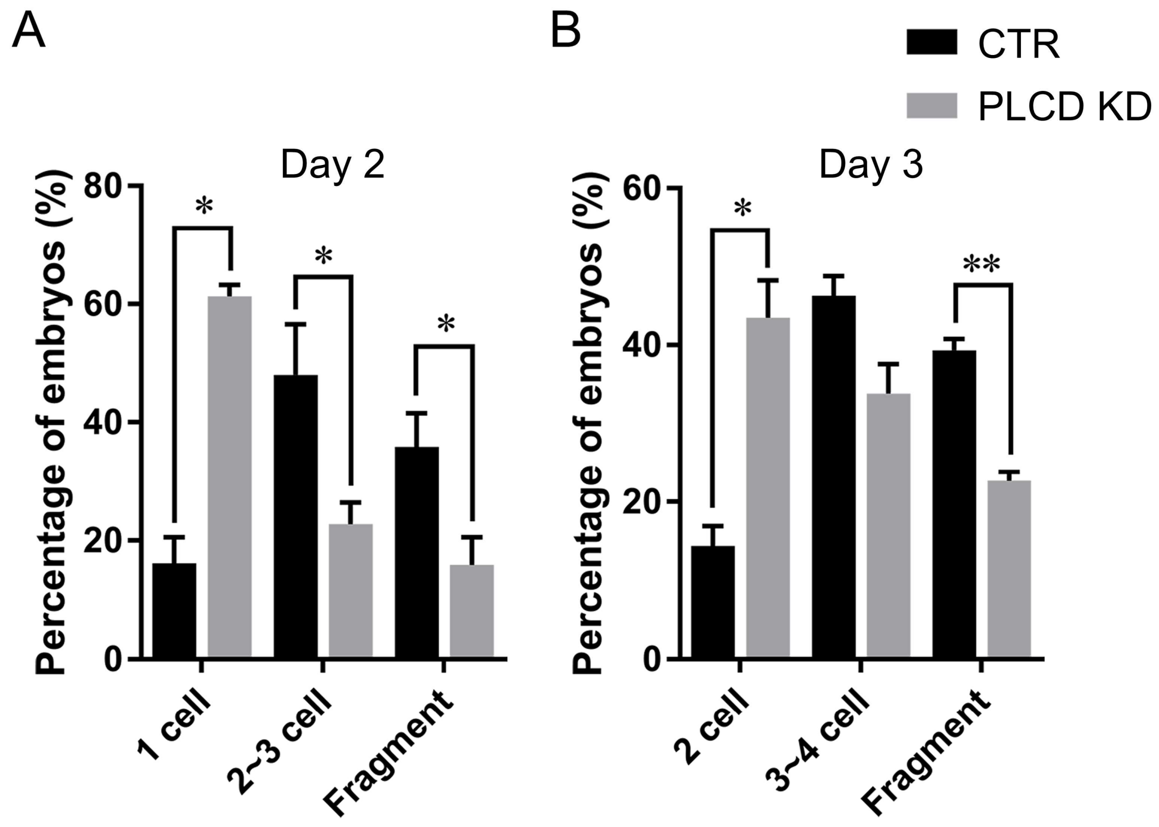

Figure S6. Effects of *PLC* knockdown on embryo development. (A) Porcine embryo development on day 2. **(B)** Porcine embryonic development on day 3.

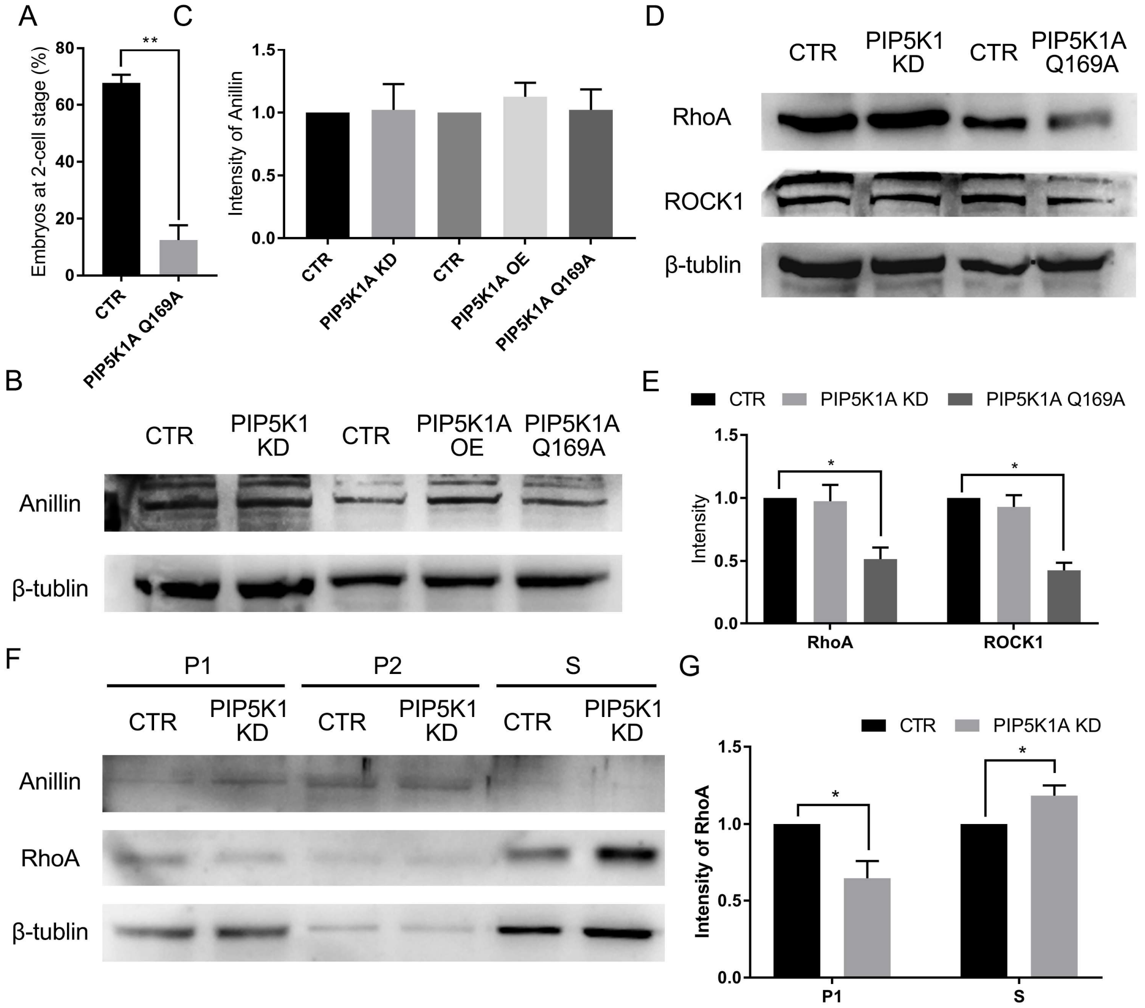

Figure S7. Regulation of PIP5K1A on RhoA signaling. (A) The ratio of embryos on day 2 after expression of the PIP5K1A^{Q169A} mutant. **(B)** Western blot for anillin in PIP5K1A KD, PIP5K1A OE, and PIP5K1A mutant groups. **(C)** Anillin protein levels after PIP5K1A knockdown, overexpression, or inactivation. **(D)** Western blot for RhoA and ROCK1 in the PIP5K1A KD and PIP5K1A mutant groups. **(E)** RhoA and ROCK1 protein levels in PIP5K1A KD and PIP5K1A mutant groups. **(F)** Western blot for anillin and RhoA in different cell fractions following PIP5K1A knockdown. **(G)** Anillin and RhoA protein levels in different cell

fractions after PIP5K1A knockdown. P1: Membranous precipitate 1. P2: Membranous precipitate 2. S: Cytoplasmic supernatant.

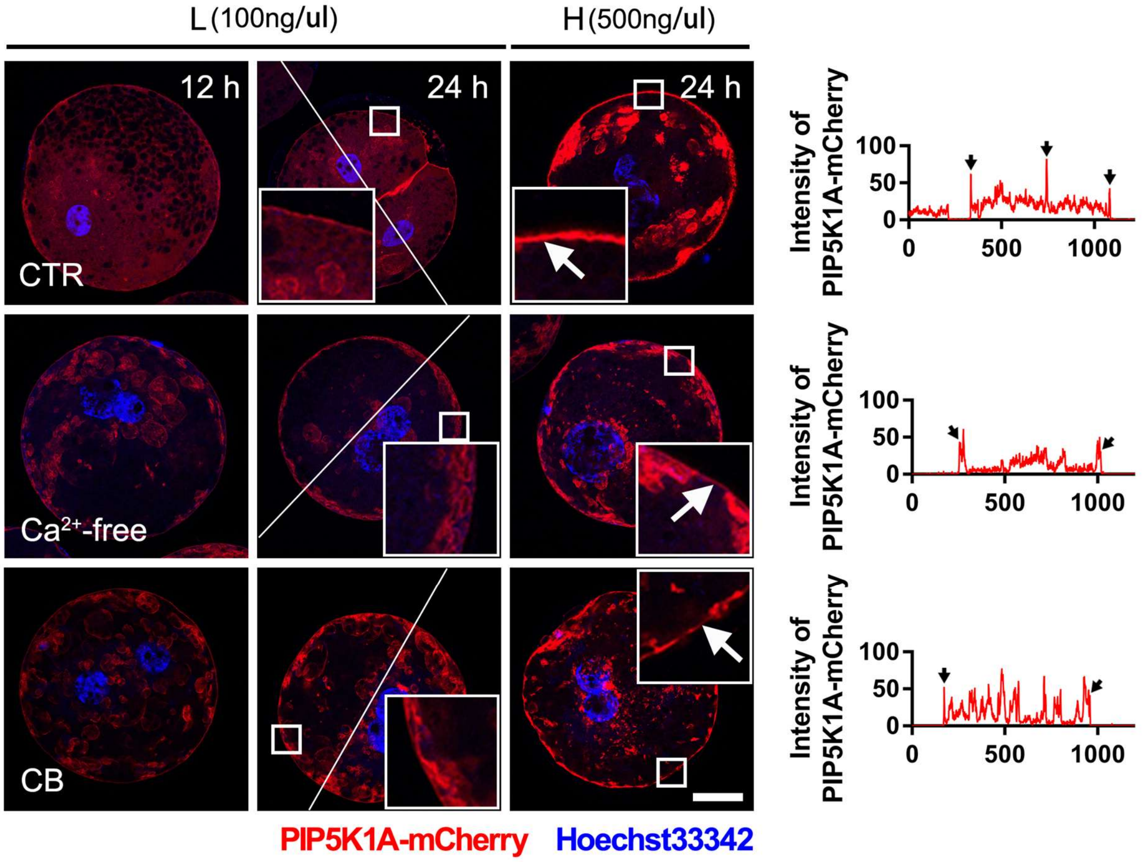

Figure S8. Localization of PIP5K1A in different treatment groups. PIP5K1A-mCherry was microinjected into 1-cell embryos, and then checked at 12 h and 24 h. L, low concentration of microinjected PIP5K1A-mCherry (100 ng/uL); H, high concentration of microinjected PIP5K1A-mCherry (500 ng/uL). The white arrow indicates the plasma membrane. In the right graphs, the intensity of PIP5K1A-mCherry was tested at the line in the L group (24 h); the dotted lines indicate the position of the plasma membrane, and the black arrow indicates the plasma membrane. Scale bar = 25 μm.

Table S1. Primers used in the RT-PCR and vector construction.

Gene name	Sequence (5’→3’)	Application
	GACAACAAAGGCGGAAGTG	RT-PCR
	GGTGAGAAATAAGGGTCAGGAAT	
	GAATTAATACGACTCACTATAGGGAGACACTA	
	CAATGACTTTCGGTTCA	Production of
	GAATTAATACGACTCACTATAGGGAGACGGCT	dsRNA
	GGATACTGTGCTT	
	CAAGTCCGGAATGGCATCTGAGGTGCTTTATG	Production of
	TGAGCTCGAGTCAGTGGATAAACTCTGACTCT	cRNA
	GTAAAAT	
	TGGACGAGCTGTACAAGTCCGGAATGCTGTGC	
PIP5K1A	TGTATGAGAAGAACCAAACAGGTTGAAAAAA	
	ATGATGAGGACCAAAAGATTATGGCATCTGAG	
	GTGCTTTATG	Production of
	CCGAGGGTTCGCGTTGAGGTTCATGTAGTATC	Q169A-mutated
	CTGG	cRNA
	AACCTCAACGCGAACCCTCGGACTTTGCTG	
	AGAATTCGAAGCTTGAGCTCGAGTCAGTGGAT	
	AAACTCTGACTCTG	
	GGCTTTGTAGGTTGAGCCGGCGAGGTCATATT	
	TGATATGCATCTTGAC	

	GCCGGCTCAACCTACAAAGCCCGGGCTTCTCA GAAAGAGC	Production of PIPB-mutated cRNA
	CAAGTTGTTAACAAACCTGTAAGACTGTAGAA TGT<br>TTACAGGTTTGTTAACAACTTGGAGCACTCTT GGAAAGC	Production of LOOP-mutated cRNA
PIP5K1B	GACCACAATTTCATCTTCTTCCTT<br>CGTCAAGCACAGAGGCATT	RT-PCR
	GAATTAATACGACTCACTATAGGGAGATCACA GAGAACCCAGACACAA<br>GAATTAATACGACTCACTATAGGGAGACGTCA AGCACAGAGGCATT	Production of dsRNA
PIP5K1C	CATGCAATGTGGAGATAGTGGT<br>GCGTCCTCCTCGTCTGAG	RT-PCR
PIP4K2A	AGGAAGAAGTGGAGTGTGAAGAG<br>GGAGCTGTTGAGCGTGTTT	RT-PCR
PIP4K2B	CCCAAGAAGGAGGTTTATTTCA<br>CAGGGTTCACAGTCGAGATTT	RT-PCR
PIP4K2C	CCCTGGAGAGTTTGAATCCTT<br>GCGTGAGCTGCCTTCTTC	RT-PCR
PTEN	AAAGACAAGGCCAACCGATA<br>GGATCAGAGTCAGTGGTGTCAG	RT-PCR

	GAATTAATACGACTCACTATAGGGAGACCCTCTTACTGCCTCCAAC<br>GAATTAATACGACTCACTATAGGGAGAGCTGTGGTGGATTATGGTCT<br>GAATTAATACGACTCACTATAGGGAGAAAAGGGACGAACTGGTGTAATG<br>GAATTAATACGACTCACTATAGGGAGACTGGATTTGATGGCTCCTCTAC	Production of dsRNAs
	CAAGTCCGGACACCTCCCGCTCCTGGAG<br>TGAGCTCGAGTCAGACTTTTGTAATTTGTGTATGCTGATCTTC	Production of cRNA
PLCD1	GACGAGCTGTACAAGTCCGGAATGGACTCAGGCCGGGACTTC<br>GAATTCGAAGCTTGAGCTCGAGTTATCACATTCCCTGAAGATCTTCCG	Production of cRNA for PH domain
PLEKHA3	GACGAGCTGTACAAGTCCGGAATGGAGGGGGTTCTGTAC<br>GAATTCGAAGCTTGAGCTCGAGTTACTAAGTCCTAGTATCAGTCAAACAGG	Production of cRNA for PH domain
GRP1	GACGAGCTGTACAAGTCCGGATTCAACCCCGACCGGGAG<br>GAATTCGAAGCTTGAGCTCGAGTCAACTGATACTGGCTCTGATGGACTTCATC	Production of cRNA for PH domain

VAMP2	TGGACGAGCTGTACAAGTCCGGAGTCGGCAACCGCTGCGAC<br>AGAATTCGAAGCTTGAGCTCGAGTCAGGGGGGAAACCTCTCCAAC	Production of cRNAs

Table S2. Concentrations of microinjected RNAs and their purposes.

Name	Concentration (ng/μL)	Purpose
PIP5K1A-mCherry cRNA	120	Localization
	200	Overexpression
	500	
PIP5K1A(Q169A)-mCherry cRNA	500	Overexpression
PIP5K1A(PIPBΔ)-mCherry cRNA	500	Overexpression
PIP5K1A(LOOPΔ)-mCherry cRNA	500	Overexpression
PIP5K1A dsRNA	1350	Knockdown
PIP5K1B dsRNA	1250	Knockdown
PTEN-mCherry cRNA	450	Localization
PTEN dsRNA	1280	Knockdown
PH_{PLCD1}*-mCherry* or *-eGFP* cRNA	550	Localization
$PH_{PLEKHA3}$*-mCherry* or *-eGFP* cRNA	450	Localization
PH_{GRP1}*-mCherry* cRNA	500	Localization
VAMP2-mCherry or *-eGFP* cRNA	370	Localization
Utrophin-mCherry cRNA	200	Overexpression
	600	
UtrCH-mCherry cRNA	560	Localization
mCherry cRNA	50	Localization
	450	Overexpression
eGFP cRNA	50	Localization
	500	Overexpression

Movie S1. Dynamics of PH_{PLCD1}-eGFP and UtrCH-mCherry in oocytes and embryos. (A) Dynamic changes of PH_{PLCD1}-eGFP and UtrCH-mCherry in oocytes and CB-treated or CB-free embryos when live imaging started at 3 h after microinjection. **(B)** Increased levels of exogenous UtrCH-mCherry induce the formation of a dynamic structure of PH_{PLCD1}-eGFP. Embryos in video Ba were obtained 3 h after they were microinjected with 100 ng/μL UtrCH-mCherry, but embryos in videos Bb and Bc were obtained 3 h and 12 h after they were microinjected with 500 ng/μL UtrCH-mCherry. Dots and bubbles in Bb exhibited polar movement toward one site on the membrane. A long time in the Bc resulted in the nucleation of large fibers with weak dynamics in PH_{PLCD1}-eGFP and UtrCH-mCherry. **(C)** Nuclei with PH_{PLCD1}-eGFP and UtrCH-mCherry formed and their polar movement toward one site at the membrane when live imaging started 6 h after microinjection. **(D)** PH_{PLCD1}-eGFP and UtrCH-mCherry aggregate at the cleavage furrow during cytokinesis. However, the cytokinesis-flattened embryos were incomplete. **(E)** Fragmented embryos exhibited phenotypes similar to cytokinesis in S1D. **(F)** Overgrowth of nuclei with PH_{PLCD1}-eGFP and UtrCH-mCherry did not trigger deepening compared with that in Movie S1D. Scale bar = 25 μm. Time showed as "minutes: seconds".

Movie S2. Effects of *PIP5K1A* knockdown on dynamics of PH_{PLCD1}-eGFP and UtrCH-mCherry. (A) Bubble formation of PH_{PLCD1}-eGFP and UtrCH-mCherry in embryos when live imaging started 3 h after microinjection. Internalized bubbles were reduced in the *PIP5K1A* knockdown group. **(B)** Formation of nuclei with PH_{PLCD1}-eGFP and UtrCH-mCherry when live imaging was initiated 12 h after microinjection. These nuclei were reduced in the *PIP5K1A* knockdown group. Scale bar = 25 μm. Time showed as "minutes: seconds".

Movie S3. Effects of exogenous *PIP5K1A* overexpression on PI4P and PIP2. (A) The dynamics of $PH_{PLEKHA3}$-eGFP in oocytes and embryos of the exogenous PIP5K1A-mCherry overexpression group. **(B)** The dynamics of PH_{PLCD1}-eGFP in oocytes and embryos of the exogenous PIP5K1A-mCherry overexpression group. **(C)** Control group for the exogenous PIP5K1A-mCherry overexpression group in videos S3A and S3B. The dynamics of $PH_{PLEKHA3}$-mCherry and PH_{PLCD1}-Egfp are shown at the interphase (Movie S3Ca) and during cytokinesis (Movie S3Cb). **(D)** Internalization of PH_{PLCD1}-eGFP from the plasma membrane into the cytoplasm, triggered by excess exogenous PIP5K1A-mCherry, resulted in the loss of contractile force, expansion of the plasma membrane, and subsequent death of the embryo. Scale bar = 25 μm. Time showed as "minutes: seconds".

Movie S4. Mutation of exogenous PIP5K1A on PIP2. (A) Dynamics of PH_{PLCD1}-eGFP in embryos of the $PIP5K1A^{K365N/K366N}$-mCherry expression group at interphase. **(B)** Dynamics of PH_{PLCD1}-eGFP in embryos of the $PIP5K1A^{K365N/K366N}$-mCherry expression group during cytokinesis. **(C)** The effect of excess $PIP5K1A^{K365N/K366N}$-mCherry on the dynamics of PH_{PLCD1}-eGFP. PH_{PLCD1}-eGFP changed from dots to bubbles, aggregated, and moved into the cytoplasm. **(D)** $PIP5K1A^{K365N/K366N}$-mCherry and PH_{PLCD1}-eGFP aggregated at one position on the plasma membrane. Scale bar = 25 μm. Time showed as "minutes: seconds".

Movie S5. Regulation of $PIP5K1A^{K365N/K366N}$-mCherry on cytokinesis. (A) $PIP5K1A^{K365N/K366N}$-mCherry aggregated in the plasma membrane during anaphase. **(B)** Aggregated $PIP5K1A^{K365N/K366N}$-mCherry initiated cleavage furrow and recruitment of the

residual spindle. **(C)** Aggregated PIP5K1A$^{K365N/K366N}$-mCherry did not capture spindles during abnormal cytokinesis. Scale bar = 25 μm. Time showed as "minutes: seconds".
